# Last Decade Insights in Exploiting Marine Microorganisms as Sources of New Bioactive Natural Products

**DOI:** 10.3390/md23030116

**Published:** 2025-03-07

**Authors:** Costanza Ragozzino, Vincenza Casella, Alessandro Coppola, Silvia Scarpato, Carmine Buonocore, Antonella Consiglio, Fortunato Palma Esposito, Christian Galasso, Pietro Tedesco, Gerardo Della Sala, Donatella de Pascale, Laura Vitale, Daniela Coppola

**Affiliations:** 1 Department of Ecosustainable Marine Biotechnology, Stazione Zoologica Anton Dohrn, Via Ammiraglio, Ferdinando Acton 55, 80133 Naples, Italy; costanza.ragozzino@szn.it (C.R.); vincenza.casella@szn.it (V.C.); alessandro.coppola@szn.it (A.C.); silvia.scarpato@szn.it (S.S.); carmine.buonocore@szn.it (C.B.); antonella.consiglio@szn.it (A.C.); fortunato.palmaesposito@szn.it (F.P.E.); pietro.tedesco@szn.it (P.T.); gerardo.dellasala@szn.it (G.D.S.); donatella.depascale@szn.it (D.d.P.); 2 Department of Chemical, Biological, Pharmaceutical and Environmental Sciences, University of Messina, Viale F. Stagno d’Alcontres, 31, 98166 Messina, Italy; 3 Department of Ecosustainable Marine Biotechnology, Calabria Marine Centre, CRIMAC, Stazione Zoologica Anton Dohrn, C. da Torre Spaccata, 87071 Amendolara, Italy; christian.galasso@szn.it

**Keywords:** natural products, marine microorganisms, metabologenomics, high-throughput screening, bioinformatic tools, bioactivity, OSMAC, co-cultivation, biotechnological applications

## Abstract

Marine microorganisms have emerged as prolific sources of bioactive natural products, offering a large chemical diversity and a broad spectrum of biological activities. Over the past decade, significant progress has been made in discovering and characterizing these compounds, pushed by technological innovations in genomics, metabolomics, and bioinformatics. Furthermore, innovative isolation and cultivation approaches have improved the isolation of rare and difficult-to-culture marine microbes, leading to the identification of novel secondary metabolites. Advances in synthetic biology and metabolic engineering have further optimized natural product yields and the generation of novel compounds with improved bioactive properties. This review highlights key developments in the exploitation of marine bacteria, fungi, and microalgae for the discovery of novel natural products with potential applications in diverse fields, underscoring the immense potential of marine microorganisms in the growing Blue Economy sector.

## 1. Introduction

Marine organisms produce high numbers of secondary metabolites, which are not essential for growth but can provide environmental advantages in specific conditions. These molecules, referred to as natural products (NPs), have been extensively exploited in several industrial fields. Over the past 60 years, more than 20,000 metabolites have been isolated from the marine environment showing antibacterial, antifungal, antiviral, antiparasitic, antitumor, anti-inflammatory, and antioxidant properties [1,2,3].

The scientific exploration of the marine environment intensified in the 1970s, with advancements in snorkeling and scuba diving, and the introduction of remotely operated vehicles (ROVs). In particular, the discovery of marine bioactive compounds started collecting organisms such as red algae, sponges, and soft corals. However, only later, it has often been shown that the actual producers of the many bioactive molecules isolated from marine invertebrates are their symbiotic microorganisms [4]. Therefore, the shift in the utilization of microorganisms is increasing, highlighting the need to overcome the problem of the sustainable supply of substances, which has caused the blocking of the commercialization of several highly promising marine compounds isolated from invertebrates. For this reason, several funded projects are concentrating on marine microorganisms (e.g., bacteria, fungi, and microalgae) for the development of new NPs, as they represent a “continuous” and still little explored resource for the discovery of new compounds [5].

Thanks to the recent development of genomic techniques and bioinformatics tools, significant advances have been made in the elucidation of microbial NP biosynthesis. The essential genes responsible for NP biosynthesis are typically organized within a contiguous segment of DNA, known as a biosynthetic gene cluster (BGC). During evolution, modular BGCs were mixed and matched, generating great structural diversity in NPs, and playing a crucial role in producing a wide range of compounds, including polyketides (PKs), non-ribosomal peptides (NRPs), terpenoids, alkaloids, and modified peptides (RiPPs, ribosomally synthesized and post-translationally modified peptides). These compounds are synthesized through complex enzymatic pathways encoded within BGCs, which contain genes for key enzymes, regulatory proteins, and transporters [6]. Moreover, these technological advancements are expanding our ability to explore untapped BGCs, even in non-culturable organisms, increasing the number of bioactive molecules discovered. The discovery of these complex biosynthetic pathways allows researchers to optimize yields, produce novel analogs, or express BGCs in heterologous hosts [7,8].

**Figure 1:**
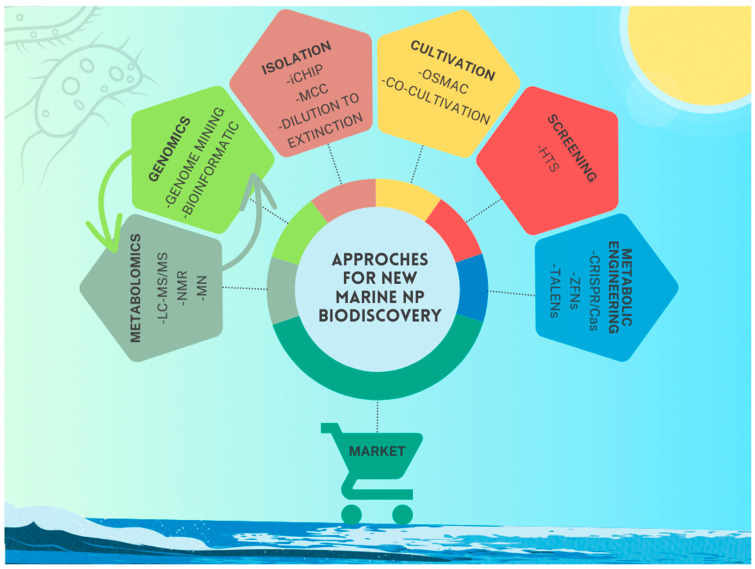
Schematic overview of innovative approaches for new natural products biodiscovery. LC-MS/MS: liquid chromatography–tandem mass spectrometry; NMR: nuclear magnetic resonance; MN: molecular networking; iCHIP: isolation chip technology, MCC: Miniaturized culture chip; OSMAC: one strain-many compounds; HTS: high-throughput screening; CRISPR: clustered regularly interspaced short palindromic repeats; ZFNs: zinc-finger nucleases; TALENs: transcription activator-like effector nucleases.

This review aims to collect the most recent data regarding the biotechnological potential of marine microorganisms, reporting the large number of new microbial NPs isolated in the last decade, with commercial potential in the blue biotechnology market. Furthermore, the review will also focus on some of the technologies used in the marine biodiscovery pipeline that have contributed significantly to the identification of this increasing number of bioactive compounds (Figure 1). In particular, some of the recent advances in technologies available for the isolation and cultivation of microorganisms will be discussed, highlighting mainly those that have contributed to increasing the exploitation of marine biodiversity and the identification of new NPs. Furthermore, considering the advances in omics technologies, alternative approaches for NP discovery were developed, compared to the classical process. In this review, we report on the recently introduced “metabologenomics”, whose strategy aims to boost the discovery of NPs by combining microbial genome mining with metabolomics [9], while recommending to refer to recent reviews [10,11,12,13] for a comprehensive overview of MS-based metabolomics. In the last decade, it is worth mentioning that molecular docking, computational methods, and cutting-edge NMR technologies have also been applied in the early screening of the NP discovery stage as well as for lead compound characterization. However, we refer readers to other comprehensive recent reviews on these topics not covered in-depth here [14,15,16,17]. Key components in the drug discovery pipeline include also high-throughput screening (HTS), which enables the rapid evaluation of bioactive compounds, and metabolic engineering, which allows for the optimization of microbial systems for the production of selected bioactive molecules, providing sustainable and scalable routes. The review will list the more recent examples where the application of these techniques led to the discovery of new marine compounds. Moreover, this review also highlights the vast commercial potential of marine microorganisms, and their diverse possible biotechnological applications, as a fundamental component of the blue biotechnology sector.

## 2. Microbial Isolation and Innovative Cultivation Strategies

### 2.1. New Techniques to Overcome the Problem of Uncultivability in Marine Microorganisms: A Special Focus on Marine Bacteria

Marine water, sediments, benthic organisms, and other organic matrices contain complex microbial populations that cooperate for physiological functions and face together environmental stimuli [18]. Despite this complexity, conventional and current laboratory techniques allow for the isolation of a small fraction of the whole microbial population, especially for bacterial species [19]. Large portions of microbial communities remain hidden for biotechnological studies and industrial applications. Isolation of a broader spectrum of new microbial species and genera is essential to expand the panel of new bioactive molecules and molecular scaffolds.

Contrarily to bacteria, uncultivability does not significantly affect the isolation of marine microalgal strains. Each microalgal species requires the optimization of growth conditions and the regulation of specific physicochemical factors (e.g., light, pH, salinity, temperature, nutrients), but isolation protocols are well optimized procedures [20]. Microalgae can be isolated in a selective or non-selective way, and the following procedures are the most standardized. Single-cell isolation can be performed using a microscope and micropipette or glass capillary. Serial dilution is a common method, among the non-selective ones, for microalgae isolation, where monoclonal purified cultures can be achieved in higher diluted samples. The agar-plating method, mostly indicated for benthic species, is another common non-selective method where agar plates with variable concentrations of nutrients are used to spread samples, and isolate morphologically different colonies [21]. Autofluorescence is an endogenous feature which represents a great advantage in microalgal isolation procedures. This can be exploited by flow cytometry and fluorescence-activated cell sorting. Flow cytometry facilities the identification of novel strains and accelerates the isolation procedure [22]. Streak plate and centrifugation (repeated low speeds cycles of centrifugation in sterile seawater) is used to increase the purity of isolated strains, since they allow for spatially separating microalgal cells from contaminating bacteria, avoiding the isolation of non-axenic cultures [23]. Also, cell sorting by flow cytometry can be a valuable and rapid tool to generate monoaxenic microalgal culture [22].

Marine fungi, although distributed in all marine habitats and involved in fundamental ecological functions, are poorly investigated in comparison to bacteria and microalgae. With the recent development of “omics” technologies, DNA sequencing of marine samples better describes the huge fungal diversity and highlights that only a few lineages have been isolated and studied so far [24]. Due to the limitation of scientific information, the portion of cultivable marine fungi is still very low and many of the protocols used for marine bacteria have been adapted to isolate fungi [25].

A very different matter is the isolation of marine bacteria. Despite enormous efforts, knowledge of bacterial physiology and adaptation processes to laboratory conditions is still poor. In nature, bacteria do not have access to high nutrient availability as in traditional growth media. Some strains start to actively grow only after a specific stimulus or unfavorable condition [26]. In addition, seasonal dynamics and ecosystem diversity strongly determine the presence and abundance of some taxonomic groups, influencing the success rate [27]. Thus, standard procedures for the isolation of bacteria have been implemented in the last decade and opportunely modified based on ecosystems of origin, taxonomic groups, and/or metabolic pathways of interest.

In this paragraph, we focus on marine bacteria, the most studied microorganisms in this sense, reviewing the publications of the last 10 years. We reported below scientific results describing new or optimized protocols that led to the isolation of rare marine bacteria, and the consequent identification of new molecules of biotechnological interest.

For example, the gelling agent in the solid media plays a key role in isolating specific marine bacteria taxa. Using gellan gum produces an increase in the viable count and recovery of bacteria that were uncultured on agar-based ones [28]. Another approach is to modify agar media preparation by reducing phosphate-catalyzed hydrogen peroxide and using scavenging agents like catalase or pyruvate to enhance colony formation [29]. Autoclaving phosphate and agar separately (PS media) improves microbial cultivability, potentially by reducing oxidative stress [30]. The “dilution to extinction” method is another technique employed in the cultivation of marine bacterial groups to estimate the number of viable microorganisms in a sample [31,32]. This approach resulted in the isolation of abundant lineages, such as SAR11 [33] and oligotrophic marine Gammaproteobacteria [31,34]. An approach used to reach the “uncultured majority” employs the diffusion chambers for in situ cultivation [35]. It is a device in which bacterial cells are cultured into a porous agar matrix within two membranes. Furthermore, the chambers are placed in the natural habitat, and the specific pore sizes act as an obstacle to cellular migration, while simultaneously allowing the diffusion of chemical growth factors, signaling molecules without direct contact with other microbes [36].

Recently, a more efficient technique that uses the iChip (isolation chip technology) multichannel device is increasing in its applicability [37,38]. This approach consists of a series of miniature diffusion chambers containing various microbial strains that are incubated in situ, increasing the cultivability rate up to 50% [39,40]. The iChip device led to the identification of the first member of a new class of antibiotics targeting lipid II, called teixobactin, from a previously uncultured Gram-negative proteobacteria *Eleftheria terrae* [41]. The isolated *Alteromonas* sp. RKMC-009 produced a new compound inhibiting the growth of *Staphylococcus aureus*. In particular, the structural elucidation using NMR spectroscopic methods identified a novel N-acyltyrosine bearing an α-methyl substituent able to inhibit the growth of *S. aureus*, and a side chain O-Methyl substituent, structural modification to the aminoacyl moiety, that increased the inhibitory activity of N-palmitoyltyrosines against enterococci [41]. The iChip platform was also successfully applied to identify *Gallaecimonas mangrovi* HK-28. This novel mangrove-derived bacterium produced three new diketopiperazines named gallaecimonamides A−C. They showed strong in vitro antibacterial activity against *Vibrio harveyi* [42]. Palma Esposito and collaborators isolated a bacterium from Antarctic Sea sediments, *Aequorivita* sp., using the Miniaturized Culture Chip (MCC) [43]. The new linear raminolipids with a N-terminal glycine produced by *Aequorivita* sp. showed anthelmintic and antimicrobial activity against Gram-positive pathogens, including Methicillin-resistant *S. aureus* DSM 18827 (MRSA) (IC_50_, 22–145 μg/mL) [43], in contrast to the pentadecylic acid that was inactive against MRSA (IC_50_, >200 μg/mL) [44].

Another isolation technique used diluted culture media together with increased incubation time. This methodology demonstrated notable success in isolating slow-growing bacteria from soil and water [45]. An example can be found in the study of Choi and collaborators [46], in which never-before-cultured bacterial taxa, belonging to the phyla Bacteroidetes and Proteobacteria, were isolated using relatively simple cultivation techniques. Marine sediments were collected from Palmyra Atoll and off Catalina Island in sterile Whirl-Pac bags at depths from <1 m to 29 m [46,47]. Isolation involved cultivating the strains on agar plates with low nutrient media and incubating them for a long time (3 weeks to 2 months) at room temperature (25 °C). Slow-growing strains with distinctive orange-yellow pigmentation were selected. Phylogenetic analysis of the 20 isolates revealed six known cultivable strains, and the identification of three new families, seven new genera, and one new species, all producing alkaloids. Antibacterial tests showed that only the CNX-216 T and CNU-914 T strains, identified in a separate study [48], exhibited notable activity. The ethyl acetate extracts resulted in the isolation of 14 alkaloid secondary metabolites across four structural classes. This included nine new compounds: marinoazepinones A and B, marinoaziridines A and B, and marinoquinolines G, H, and I from strain CNX-216T, along with marinopyrazinones A and B from both strains CNX-216T and CNU-914T [48].

I-tip (in situ cultivation by tip) was determined to be a highly effective strategy for isolating new bacteria associated with marine sponges. This method allows for the isolation of invertebrate-associated microorganisms (symbionts) using standard tip micropipettes placed on the organisms and filled with agar and microbeads. In fact, the study of Jung and collaborators points out a higher diversity and richness of species isolated from Baikalian sponges with the I-tip method compared to the conventional cultivation method [49]. This technique is very prolific for some phyla but not favorable for all others. However, in situ cultivation methods, thanks to the presence of growth initiation factors (signaling-like compounds), allowed for the isolation of sponge-associated bacteria that were considered previously non-growing using standard cultivation methods [50].

Furthermore, the addition of antibacterial and antifungal agents into the isolation media significantly enhances the selective isolation of different rare Actinomycetota [51], which are known to secrete antibiotics, antiproliferative, antimalarials, and immunosuppressive molecules [52]. An additional approach involved the addition of inhibitors against Gram-negative bacteria and fungi into the media [53], like nalidixic acid and quinolone antibiotics [54]. Moreover, achieving successful isolation of unknown strains demands expertise in Actinomycetota physiology and taxonomies [55]. Actinomycetota, especially soil-derived species of the genus *Streptomyces*, are known for producing a wide range of bioactive metabolites [56], although marine strains have shown significant potential as sources of new bioactive compounds; examples include antimalarials like salinipostins, cytotoxics like marinomycins, and antibacterials like abyssomycins, isolated from marine environments [57,58,59]. An example is the genus *Nocardiopsis*, which is mainly present in extreme habitats and is, therefore, very hard to culture [60]. In the study of Siddarth, researchers explored marine sediments from Havelock Island, Andaman, and Nicobar Islands; the samples were plated on starch casein agar (SCA) with cycloheximide and nalidixic acid. This led to the isolation of *Nocardiopsis* sp. SCA21, producing two new bioactive compounds in liquid culture: 4-bromophenol and Bis(2-ethylhexyl) phthalate. Both compounds exhibited broad-spectrum antagonistic activity against Gram-positive and Gram-negative pathogens, with potent antibacterial efficacy. Additionally, they demonstrated remarkable antioxidant potential, scavenging DPPH and ABTS radicals, while the metal chelating activity was less significant [61]. Isolated from the shores of the Caspian Sea in Iran, the unusual Actinomycetota *Saccharothrix xinjiangensis* Act24Zk led to the discovery of three novel compounds: caerulomycin M, saccharopyrone, and saccharonic acid. Notably, saccharopyrone displayed moderate cytotoxic activity against human cervical carcinoma cells HeLa KB-3-1, with an IC_50_ value of 5.4 µM [62].

New compounds derived from marine bacteria, obtained through the application of innovative isolation techniques, are listed in Table 1. However, continual refinement and improvement of isolation procedures are necessary, making the isolation of rare bacteria an ongoing and limitless process.

### 2.2. Microbial Cultivation Approaches to Harness Untapped Biosynthetic Potential

More than half of the drugs used in the clinical field are NPs of microbial origin, generally encoded by different BGCs. However, finding new microbial NPs is difficult, mainly because of the high rediscovery rate of known compounds [63,64]. Among the possible causes, there are difficulties in finding the ideal culture conditions for the expression of relevant BGCs, not expressed under traditional cultivation conditions [65]. Different approaches and cultivation strategies were successfully applied to unlock the unexplored biosynthetic potential of marine microorganisms.

Among the culture-dependent methods used to induce the activation of microbial cryptic BGCs, the “One Strain—MAny Compounds” (OSMAC) strategy is the most successful; it emphasizes how a single species can produce different molecules when the cultivation parameters are changed [66]. This approach consists mainly in the variation in growth factors affecting microbial cultivation (concentration and source of nutrients in growth media, temperature, pH, osmotic conditions, aeration), along with the addition of inducer molecules (metals, vitamins, and other oligoelements) that can trigger the production of metabolites unexpressed or under-expressed by traditional cultivation approaches [66,67]. The use of liquid or solid media in cultivation is also crucial, as well as the type of recipient, the aeration, and the agitation conditions [68,69].

In addition, another efficient technique used to stimulate the production of NPs and the activation of “silent” BGCs is the co-cultivation of different microorganisms [70,71]. The co-cultivation (also called mixed fermentation) of two or more microorganisms in the same confined environment often leads to interesting outcomes, such as the enhanced production of known compounds or the discovery of cryptic compounds that may not be detected in axenic cultures [70,72,73]. When microorganisms are grown together in co-culture, they interact in various ways, as in nature, including competing for resources, exchanging signal molecules (quorum sensing), adhesion factors (biofilms), and displaying cooperative metabolic activities [74,75]. Various strategies have been implemented, including the adoption of a communal liquid medium for growth, the use of a solid–liquid interface (bead entrapment) [76], cell droplets [77], separation through membranes [78], spatial arrangement (the physical arrangement of microorganisms in distinct spaces, favoring indirect interactions), and the use of microfluidic systems [79]. In addition, some strategies involve selecting microbes from the same environment or combining cultures of microorganisms that are unlikely to naturally coexist [80]. Mixed fermentation is a good strategy to solve the problem of monoculture contamination of microalgae, which leads to low productivity in terms of biomass and compounds compared to co-cultures with other microorganisms [81,82,83]. Microalgae–bacteria consortia proved to be more advantageous and efficient due to the synergistic interactions they establish, including nutrient cycling and organic matter decomposition (dissolved organic matter—DOM) [84,85,86].

**Figure 2:**
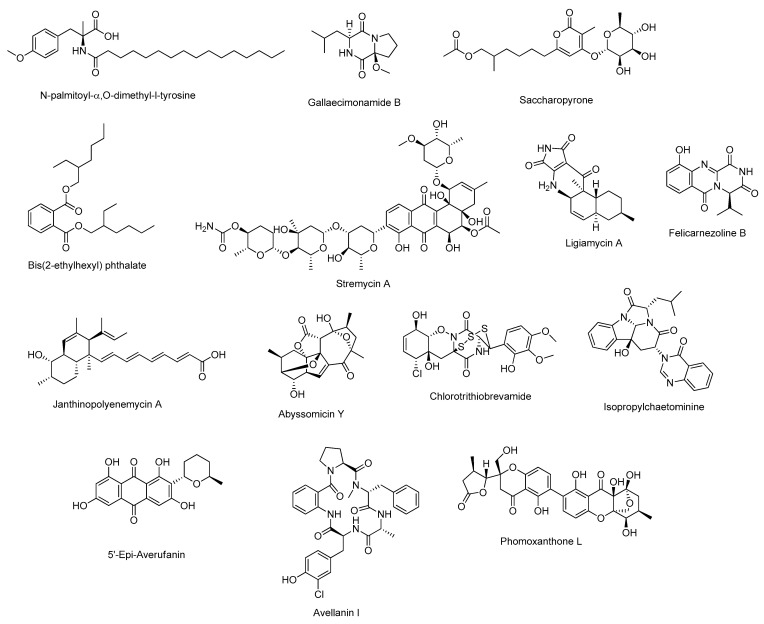
Chemical structure of most promising novel natural products isolated through innovative isolation approaches and new cultivation techniques from marine-derived microorganisms.

During recent years, by these “non-traditional” cultivation approaches, numerous novel bioactive molecules have been discovered from marine microorganisms, which are listed in Table 1; in the following paragraphs, some significant examples arising from these approaches will be discussed in detail. Moreover, the chemical structure of the most promising novel natural products of Table 1 are shown in Figure 2.

**Table 1:** List of novel natural products isolated through innovative isolation approaches and new cultivation techniques from marine-derived microorganisms.

Microorganism	Origin	Place	Class	Compounds	Innovative Strategies	Bioactivity	Assay	Activity Value	References
**BACTERIA**									
*Alteromonas* sp. RKMC-009	Sponge (*Xestospongia muta*)	San Salvador, The Bahamas	N-acyltyrosine	N-palmitoyl-α,O-dimethyl-l-tyrosine	iChip	Antibacterial	Microdilution broth	IC_50_ < 8 μM vs. *Enterococcus* strains	[87]
*Gallaecimonas* *mangrovi* HK-28	Mangrove sediment	Hainan province, China	Diketopiperazines	Gallaecimonamides A–C	iChip	Antibacterial (Gallaecimonamides B)	Microdilution broth	MIC 50 μM vs. *V. harveyi*	[42]
*Mooreia alkaloidigena* CNX-216T *Catalinimonas alkaloidigena* CNU-914T	Marine sediment	Palmyra Atoll, USA	Alkaloids	Marinoazepinone A–B; Marinoaziridine A–B; Marinoquinolines G–I, Marinopyrazinones A	Low-nutrient media and extended incubation times	Antibacterial	Agar diffusion disk	Inhibiton diameter ranging from 9 to 18 mm vs. *Pontibacillus* sp.	[48]
				Marinopyrazinones B				14 mm of inhibiton diameter vs. *Pontibacillus* sp., 9 mm vs. *Vibrio shiloi*	
*Nocardiopsis* sp. SCA21	Marine sediment	Havelock Andaman Nicobar Islands	Not reported	4-bromophenol (1), Bis(2-ethylhexyl) phthalate (2)	Addition of inhibitors into the media	Antibacterial, antioxidant, and metal chelating activity	Microdilution broth, DPPH, ABTS Ferrozine assay	MIC ranging from 7.81 to 250 μg/mL vs. *Bacillus subtilis* ATCC 6633, *Klebsiella pneumoniae* ATCC 13883, *Listeria cytogens* ATCC 13932, *Salmonella typhi* ATCC 25241, *Staphylococcus aureus* ATCC 12600, MRSA ATCC NR-46071, and MRSA ATCC NR-46171; DPPH IC_50_ values 187.31 μg/mL (1), 96.57 μg/mL (2); ABTS 102.22 μg/mL (1), 151.60 μg/mL (2) Ferrozine assay 19.41% at 31.25 μg/mL and 62.89% at 1 mg/mL (1) 56.10% at 1 mg/mL (2)	[61]
*Saccharothrix xinjiangensis* Act24Zk	Marine sediment	Caspian Sea, Iran	PKS	caerulomycin M; saccharopyrone; saccharonic acid	Isolation method for myxobacteria	Antiproliferative and antibacterial	MTT and microdilution broth	MTT IC_50_ ranging from 0.1 to 50.7 µM vs. L920 and KB3.1 MIC ranging from 4.2 to 66.7 µg/mL vs. *Staphylococcus aureus* Newman, *Micrococcus luteus* DSM1790, *Escherichia coli* DSM1116, *Mucor hiemalis* DSM2656, and *Candida albicans* DSM1665	[62]
*Streptomyces pratensis* NA-zhous1	Marine sediment	Zhoushan Coast, China	Angucycline (PKS)	Stremycin A–B	OSMAC	Antibacterial	Microdilution broth	MIC 16 µg/mL vs. MRSA, *K. pneumoniae*, *E. coli* (Stremycin A-B) and vs. *B. subtilis* (8 µg/mL Stremycin A and 16 µg/mL Stremycin B)	[88]
*Janthinobacterium* spp. ZZ145 *Janthinobacterium* spp. ZZ148	Marine sediment	Sindh, Karachi, Pakistan	PKS	Janthinopolyenemycins A–B	Co-cultivation	Antifungal	Microdilution broth	MIC 15.6 μg/mL vs. *C. albicans*	[89]
*Verrucosispora* sp. MS100137	Deep-sea sediment	South China Sea	PKS	Abyssomicin Y	OSMAC	Antiviral	Cell viability	98% of influenza A virus inhibition at 10 μM	[59]
*Saccharothrix* sp. D09	Intertidal zone sediment	Fushan Bay, Qingdao, China	PKS	Saccharotrins D–I	OSMAC	Antibacterial (F–G); antioxidant (F)	Microdilution broth	MIC 32 μg/mL vs. *H. pylori* 159; IC_50_ 28 μM	[64]
				Glycosylated saccharotrixines J–M		Antibacterial (K)		MIC 16 μg/mL vs. *H. pylori* G27, *H. pylori* 159, and *S. aureus* ATCC25923	
*Streptomyces* sp. GET02.ST *Achromobacter* sp. GET02.AC	Marine Wharf Roach (*Ligia exotica*)	Seocheon, Chungcheongnam-do, Korea	PKS-NRPS	Ligiamycins A–B	Co-cultivation	Antibacterial	Microdilution broth	(A) MIC 16 μg/mL vs. *S. aureus* ATCC25923 and *S. enterica* ATCC14028 (B) MIC 64 μg/mL vs. *S. aureus* ATCC25923	[90]
*Streptomyces griseorubiginosus*	Cnidarian	Réunion Island, France	Aromatic PKS	Five new aromatic PKS (19–23) differing in novel anthrone backbones, a naphtho[2,3-c]furan-4(9H)-one chromophore group and novel tetralone structure	OSMAC	Antibacterial; cytotoxic	Not reported	No activity	[91]
*Microbacterium* sp. V1	Marine sponge (*Petrosia ficiformis)*	Ischia Island, Italy	Peptide	Two proline-rich peptides	OSMAC	Antioxidant	FRAP	FRAP from 51 to 121 μM	[92]
	**FUNGI**								
*Thrichoderma* sp. TPU199	Red alga	Palau	Diketopiperazine	Chlorotrithiobrevamide	OSMAC	Antiproliferative	MTT	IC_50_ 16 μM vs. T-cell leukemia Jurkat cells	[93]
*Aspergillus sclerotiorum* SCSGAF 0053 *Penicillium citrinum* SCSGAF 0052	Gorgonian (*Muricella flexuosa*)	Hainan, South China Sea	Furanone and oxadiazin derivatives	Sclerotiorumins A–C 4-benzyl-1H-pyrrol-3-yl Aluminiumneohydroxyaspergillin Ferrineohydroxyaspergillin	Co-cultivation	Cytotoxic (Aluminiumneohydroxyaspergillin)	MTT	IC_50_ 4.2 μM vs. U937	[94]
*Aspergillus carneus*	Sponge (*Agelas oroides*,)	Aegean Sea coast, Turkey	Quinazolinone and anthraquinone derivatives	Isopropylchaetominine, Isoterrelumamide A, 5′-Epi-Averufanin	OSMAC	Cytotoxic (Isopropylchaetominine)	MTT	IC_50_ 0.4 μM vs. L5178Y	[95]
						Antibacterial (5′-Epi-Averufanin)	Microdilution broth	MIC 4.63 μg/mL vs. *S. aureus* ATCC 700699 and 9.3 μg/mL vs. *E. faecium* ATCC 35667	
*Asteromyces cruciatus*	Sea foam	Point Prim, Prince Edward Island, Canada	PKS	Primarolides A–B	OSMAC	Antimicrobial	Microdilution broth	No activity	[96]
*Fusarium equiseti* D39	Plant	Intertidal zone of the Yellow Sea, Qingdao, China	3-decalinoyltetramic acid derivatives	Fusarisetins B–D	OSMAC	Phytotoxicities	Phytotoxicity	200 μg/mL vs. growth of amranth	[97]
*Penicillium restrictum* MMS417.	Blue mussel (*Mytilus edulis*)	Loire estuary, France	Pyranone derivatives	Five pyranone derivatives	OSMAC	Antimicrobial	Paper disk diffusion	No activity	[98]
						Cytotoxic	MTT	Not cytotoxic Vs. KB at 50 μg/mL	
						Antileishmanial activity	Luminescence	No activity	
*Aspergillus* sp. SCSIO 41501	Gorgonian (*Melitodes squamata*)	Hainan, South China Sea	Cyclopentenone and cyclohexenones derivatives	Aspergispones A–H	OSMAC	Not reported	Not reported	No activity	[99]
*Penicillium* sp. HLLG-122	Root of mangrove (*Lumnitzera littorea*)	Sanya, Hainan Island, China	Meroterpenoids	Peniciacetals A–I	OSMAC	Cytotoxic	Celltiter-glo Kit	(E,F) IC_50_ < 25 μM vs. HepG2 (B,E,F) IC_50_ < 26 μM vs. MCF-7	[100]
*Phomopsis* sp. QYM-13	Leaves of mangroove (*Kandelia candel*)	Hainan Province, South China Sea	Cytochalasins	Phomopchalasins D–O	OSMAC	Cytotoxic	MTT	IC_50_ < 50 μM vs. MDA-MB	[101]
*Alternaria alternata* LW37	Deep-sea sediment	Southwest Indian Ridge	Dibenzo-α-pyrone derivatives	Alternolides A–C	OSMAC	α-Glucosidase inhibition (C)	α-Glucosidase Inhibition	IC_50_ of 451.25 μM	[102]
*Beauveria felina* KMM4639 *Aspergillus carneus* KMM4638	Brown alga (*Laminaria sachalinensis*)	Kunashir Island, Russia	Sesquiterpenes	Asperflavinoids B, D, and E	Co-cultivation	Cytotoxic	MTT	IC_50_ ranging from 51 to 95 μM vs. PC-3, MCF-7, Raji cells, H9c2	[103]
*Aspergillus versicolor* PS108-62	Deep-sea sediment	Farm Strait, Arctic Ocean	PKS-NRPS Macrolactone	Versicolide A, isoversicomide A	OSMAC	Not reported	Not reported	Not reported	[104]
*Phomopsis lithocarpus* FS508	Not reported	Not reported	Benzoic Acid derivative	4-methoxy-3-[4-(acetyloxy)-3-methyl-2-butenyl]benzoic acid	OSMAC	Not reported	Not reported	Not reported	[105]
*Emericellopsis maritima* BC17	Marine sediment	Bay of Cádiz, Spain	Eremophilanes	Four new eremophilane-type sesquiterpenes	OSMAC	Antimicrobial antiproliferative	Microdilution broth MTT	Not detected	[106]
*Cosmospora* sp. *Magnaporthe oryzae*	Windebyer Noor	German Baltic coast	Isochromanones	Soudanones H–I	Co-cultivation	Not reported	Not reported	Not reported	[107]
*Aspergillus fumigatus* KMM 4631 *Penicilliumhispanicum* KMM 4689	Coral (*Sinularia* sp.)	Kunashir Island, north west Pacific Ocean	Not identified	Two unidentified novels compounds	Co-cultivation	Antioxidant cytotoxic urease inhibition	DPPH∙ MTT indophenol method	53% of scavenging effect and 9% of urease inhibition at 100 µg/mL; 10% of viability reduction at 10 µg/mL vs. HepG2	[108]
*Amphichorda* KMM4639 *Aspergillus carneus* KMM4638	Brown alga (*Laminaria sachalinensis*)	Kunashir Island, north west Pacific Ocean	Quinazolinone alkaloids Chromene derivative	Felicarnezolines A–E Oxirapentyn M	Co-cultivation	Cytoprotective against CoCl_2_ (Felicarnezoline B)	MTT	72.6% and 19% increased viability of SH-SY5Y and H9c2, respective ely	[109]
*Aspergillus insulicola* IMB18-072 *Alternaria angustiovoidea* IMB20-805	Unidentified marine sponge	Weizhou Island, China	Cyclic tetrapeptides	Violaceotides B–E	Co-cultivation	Antimicrobial	Microdilution broth	(B–C) MIC values of 64–128 μg/mL vs. aquatic pathogens *Edwardsiella tarda* ATCC15947, *E. tarda* QDIO-2, and *E. ictaluri* ATCC33202	[110]
*Phomopsis asparagi* DHS-48 *Phomopsis* sp. DHS-11	Root of mangrove (*Rhizophora mangle)*	Dong Zhai Gang-Mangrove Garden on Hainan Island, China	Alkaloids, sterols, polyketides	Phomopyrazine, phomosterol C, phomopyrone E	Co-cultivation	Cytotoxic	MTT	IC_50_ value 65.97 μM vs. HepG2 and 72.02 vs. Hela	[111]
						Immunosuppressive	Splenocyte proliferation assay	(Phomopyrazine) IC_50_ 125.1 and 133.87 μM against the proliferation of ConA-induced (T-cell) and LPS-induced (B-Cell), respectively	
						Acetylcholinesterase (AchE) inhibitory activities		No activity	
*Phomopsis asparagi* DHS-48 *Phomopsis* sp. DHS-11	Root of mangrove (*Rhizophora mangle)*	Dong Zhai Gang-Mangrove Garden on Hainan Island, China	Dimeric xanthones	Phomoxanthones L–N	Co-cultivation	Cytotoxic	MTT	(L-N) IC_50_ values 53.72, 50.25 and 67.32 μM vs. HepG2 (L-N) IC_50_ values 69.53, 67.66 and 87.32 vs. Hela	[112]
						Immunosuppressive	Splenocyte proliferation assay	(L-N) IC_50_ 55.53, 60.25, and 75.75 μM against the proliferation of ConA-induced (T-cell) (L-N) IC_50_ 89.27, 87.66 and 102.65 against LPS-induced (B-Cell)	
*Aspergillus alabamensis* SYSU-6778 *Aspergillus fumigatiaffinis* SYSU-6786	Roots of seagrass (*E. acoroides)*	Hainan Island, the People’s Republic of China	Chromone, benzoic acid derivative	(10) (2*R*, 3*R*)-5,7-dihydroxy-2,3-dimethyl-4-chromanone, (13) 5-hydroxy-3,4-dimethoxy-2-methylbenzoic acid	Co-cultivation	Antifungal	Microdilution broth	(10, 13) MIC 100 μg/mL and >200 μg/mL against *A. alabamensis* SYSU-6778	[113]
*Eutypella* sp. D-1	Marine sediment	London Island of Kongsfjorden in the Ny-Ålesund, Arctic Ocean	Cytosporin polyketides	eutypelleudesmane A, cytosporin Y, cytosporin Z, cytosporin Y_1_, cytosporin Y_2_, cytosporin Y_3_, and cytosporin E_1_	OSMAC	Antimicrobial	Microdilution broth	MIC > 128 μg/mL vs. *S. aureus*, *E. coli*, *B. subtilis*	[114]
						Cytotoxic	CCK-8	IC_50_> 50 μM vs. DU145, SW1990, Huh7, and PANC-1	
						Immunosuppressive	calcineurin (CN) phosphatase assays	(Z,Y_3_) 62.9% and 59.5% against ConA-induced T cell proliferation, respectively, at a concentration of 5 μg/mL	
*Penicillium velutinum* ZK-14	Seagrass (*Zostera marina*)	Sea of Japan, Japan	2,5-dibenzylpiperazine	Helvamides B,C	OSMAC	Cytotoxic	MTT	(B) 34.03% and 16.64% (C) 39.41% and 28.42% decreased viability of PC-3 and HEK-293, respectively	[115]
						Antifungal	Microdilution broth	(B) 7% and (C) 31,54% of inhibition of *C albicans*	
						Antioxidant	DPPH	4.92% and 4.5%, respectively	
*Hamigera ingelheimensis* MSC5	Anemone	Mariana Trench, Pacific Ocean	NRPS	Avellanins D–O	OSMAC	Antimalarial activity	Not reported	(D, E, F, H, I) IC_50_ values of >50 μM, 16 μM, 10 μM and 0.19 μM vs. *P. falciparum* 3D7, respectively.	[116]
						Cytotoxicity	MTT	No activity vs. A549, MKN-45, HCT-116, HeLa, K562, 786-O, TE-1, 5637, GBC-SD, MCF-7, HepG2, SF126, DU145, GAL-62, PATU8988T, HOS, A-375, A-673, L-02, and 293T	

MTT: thiazolyl blue tetrazolium bromide; FRAP: ferric-reducing ability of plasma, DPPH: 2,2-difenil-1-picrylidrazyl radical; PKS: polyketide synthase; NRPS: non-ribosomal peptide.

#### 2.2.1. One Strain-MAny Compounds (OSMAC) in Marine Microbial Drug Discovery

The success of the OSMAC technique applied primarily to terrestrial microorganisms has gradually found its way into the marine world, as demonstrated by the numerous research articles published during the last 10 years. In the review by Romano et al. [71], the authors discussed most of the parameters successfully affecting the OSMAC strategy when applied to marine microorganisms, reporting works from 2002 to 2018. Similarly, Pan et al. reviewed the application of the OSMAC approach on terrestrial and marine species, specifically focusing on the chemical and structural diversity of produced secondary metabolites [117]. More recently, Pinedo-Rivilla and co-authors [118] examined the structures and biological activities of recently discovered cryptic metabolites from marine-derived microorganisms obtained through the OSMAC approach, especially employing epigenetic modifiers among other approaches [118]. Many reviews discuss this strategy associated with the identification of new bioactive compounds [119], although, for microalgae, the OSMAC technique is commonly used to optimize and enhance the yield of specific compounds [120,121,122].

Since only some of the new marine compounds obtained in the last 10 years through this strategy have been reviewed [71,117], herein we decided to complement the current reviews by describing the molecules not previously reported (Table 1).

For example, following the OSMAC strategy, the marine isolate *Rhodococcus* sp. I2R was cultivated in 22 growth conditions, differing in nutrient sources and including very rich and minimal media [67]. Notably, only the carbon-rich SV2 SW medium, supplemented with glycerol, triggered the production of a novel glycolipid mixture, composed of novel succinoyl trehalolipids bearing an unusual phenylacetate unit. The new compounds demonstrated to possess potent antiviral activity against enveloped viruses, moderate in vitro antiproliferative effects toward human prostate cancer cell line (PC3), and biosurfactant activity. Using the same approach, the marine-derived strain *Streptomyces* sp. RKND004 was grown in 14 different liquid media. Chemical screening led to the selection of the BFM3 media (MgSO_4_·7H_2_O, KCl, K_2_HPO_4_·3H_2_O, NaCl, Nutrient agar, Glycerol, Bacto soytone) for large-scale bacterial fermentation, thus enabling the isolation of two known terrosamycins (A–B), which showed to be potent antimicrobials and anticancer agents against HTB-26 and MCF-7 breast cancer cell lines [123].

Shen et al. applied this strategy to a rare marine Actinomycetota *Saccharothrix* sp. D09, leading to the discovery of 20 angucycline derivatives, including 6 novel highly oxygenated saccharothrixins D–I, and four new glycosylated saccharothrixines J–M, together with 10 known analogs. Saccharothrixins F, G, and K showed growth inhibitory activity against *Helicobacter pylori*, and saccharothrixin F also inhibited NO production induced by LPS in RAW 264.7 macrophages [124].

Within the phylum Actinomycetota, *Micromonospora* spp. are known for their ability to produce a wide variety of bioactive secondary metabolites, including antibiotics, antifungals, anticancer agents, and immunosuppressants [125]. A novel PK belonging to the abyssomycin family, abyssomicin Y, was isolated using the OSMAC method from the marine-derived strain *Verrucosispora* sp. MS100137, together with six known abyssomicin and proximicin analogs. The novel compound displayed potent anti-influenza A virus activity [59]. The research group of Kokkini reported *Micromonospora* marine species as the best producers of phocoenamicins, as a result of an HTS based on the OSMAC approach, which involved the exploration of 27 actinomycete strains, including three marine species, cultivated in 10 different media. Combining metabolomic analysis and molecular networking of the 270 crude extracts revealed the presence of phocoenamicins, with phocoenamicin A being the most abundant compound produced in all tested strains and conditions, while phocoenamicin B and C were produced only in RAM2-P V2, FR23, M016, and SAM-6 media. In addition, the influence of five parameters (different strains, growth media, taxonomic species, ecological and geography sources) on metabolite production was evaluated, observing that the culture medium has the greatest impact on the variation in the metabolic profile. The evaluation of the antimicrobial activity of the phocoenamicins A-C revealed that the media richer in carbon were better performing than the poorer ones [126]. Similarly, the influence of media components, culture support (solid–liquid), and incubation times were evaluated for two marine strains of *Micromonospora* (SH-82 and SH-57) and *Salinispora arenicola* (SH-78), isolated from the marine sponge *Scopalina hapalia* (ML-263). The solid support was found to be optimal in metabolic diversity and activity for the SH-82 strain, while liquid culture was better for the SH-57 and SH-78 strains, confirming that the influence of culture parameters depends on the species studied and affects microbial metabolites production [127].

Regarding sponge-associated Actinomycetota, several researchers applied the OSMAC approach to select the optimal medium for the production of new bioactive compounds. Among three different culture media, the International Streptomyces Project-2 (ISP2) medium was the best for the production of bioactive metabolites, displaying antimalarial activity against the strain *Plasmodium falciparum* by *Streptomyces* sp. US4 [128]. The Abdelaleem’s work showed that, among different media used to evaluate the bioactive potential of various strains isolated from *Spongia irregolaris*, only two unlocked the production of compounds with anti-HCV activity, i.e., ISP2 and oligotrophic (OLIGO) media for *Micromonospora* sp. UR44, and ISP-2 for *Streptomyces* sp. UR32. [129]. Through eight different conditions, combining four media and two temperatures, it was shown that the low temperature expanded the chemodiversity and activated the transcription of *Microbacterium* sp. V1, producing two antioxidant proline-rich peptides with an unprecedented planar structure containing a N-terminal pyroglutamic acid [92].

The OSMAC strategy, combined with an untargeted metabolomics approach, is often used in the first phase of NP discovery together with functional screenings, facilitating the subsequent isolation of pure bioactive metabolites. The efficacy of this combination was crucial in the first study of the bacterial community associated with the mesopelagic jellyfish *Periphylla periphylla*, using two different culture media, which revealed a high antimicrobial potential [130]. The study by Soldatou et al. demonstrated the effectiveness of the OSMAC strategy combined with an innovative metabolomics approach in discovering new bioactive metabolites from 25 polar Actinomycetota. The researchers found that the use of different growth media generated a wide range of metabolites, with 72% of them showing activity against six resistant pathogenic bacteria, including *Klebsiella pneumoniae* and *Acinetobacter baumannii* [131].

As reported by the review of Pinedo-Rivilla and co-authors, the OSMAC strategy also includes the addition of small molecules to the culture media, such as chemical elicitors, which influence gene expression at the transcriptional level [118].

For example, de Felicio and collaborators performed a screening carried out on marine bacteria by the supplementation of different elicitors: procaine and sodium butyrate (chemical elicitors); ampicillin, kanamycin, chloramphenicol, and streptomycin sulfate (natural antibiotics); and DMSO and EDTA (organic solvent). Analyzing the elicitors effects through non-targeted metabolomics, the results demonstrated the production of a substantial portion of new, rare, and bioactive secondary metabolites [132]. More recently, the marine *Streptomyces griseorubiginosus*, derived from an unidentified cnidarian isolated from a reef in Réunion Island (France), was cultivated by using the rich medium FA-1 in the presence and absence of the chemical elicitor, γ-butyrolactone. This fermentation led to the synthesis of several known products not expressed under standard conditions, and the isolation of five different new aromatic PKs holding a novel anthrone backbone, distinctive tetralone structure, and unusual naphtho [2,3-c]furan-4(9H)-one chromophore, along with additional known compounds. However, none of them exhibited antibacterial and cytotoxic effects against various bacteria and tumor cell lines [91]. A multifactorial OSMAC approach was performed for the myxobacterium *Corallococcus coralloides*, possessing many BGCs coding for potential new NPs. A minimal and a complex medium were tested simultaneously with biotic additives and organic solvents as elicitors. The synergy of these bivariate OSMAC experiments led to the detection of 55 completely new molecules, compared to those produced by univariate screening with one elicitor. This showed that the exposure of microorganisms to many different stimuli is necessary to induce the activation of cryptic biosynthetic pathways and fully express the biosynthetic potential [133].

Similarly to bacteria, the application of the OSMAC strategy to marine fungi led to the discovery of a wide spectrum of secondary metabolites, highlighting their biodiversity, ecological roles, and biotechnological potential. For example, the use of the untargeted metabolomic approach combined with OSMAC strategy was useful in assessing the antitumor potential of the fungal community from Quanzhou Bay (China), in which 77 crude extracts displayed different antiproliferative activities from the same strain grown under different conditions [134]. Le V-T et al. showed how the media composition could influence the growth and by-products of the *Penicillium restrictum* MMS417 strain, a fungus isolated from the blue mussel *Mytilus edulis* collected on the Loire estuary, France. Using an untargeted approach, the authors cultivated the fungal strain under 14 different culture conditions to evaluate the effects of the mussel flesh on the metabolomic profile. The mussel-derived medium with synthetic seawater induced the overexpression of specific compounds and led to the isolation of 12 piran-2-ones, including five new pyran-2-one derivatives. This work demonstrated that the simulation of the natural environment, through the use of the combination of seawater and host-derived substrates in the medium, allows for the production of new metabolites [98].

A cooperative induction strategy was used by Igboeli and collaborators for the cultivation of the marine fungus *Asteromyces cruciatus* through both epigenetic modifications and osmotic stress, with suberoylanilide hydroxamic acid and sodium chloride. In addition to a greater chemodiversity of compounds produced, this abiotic stress induced the synthesis of two new PKs. Primarolides A and B exhibited discrete antimicrobial activity against *S. aureus* and *Candida albicans* [96]. Moreover, the marine fungus *Thrichoderma* sp. TPU199 grown in a seawater-based natural medium, supplemented with DMSO (1% final concentration), produced a new compound, namely chlorotrithiobrevamide [93].

Bioactive fungi have been isolated from a wide range of marine or transitional environments. For example, the mangrove-derived fungus *Phomopsis* sp. QYM-13 was cultivated using an OSMAC approach, enriching the potato liquid medium with 3% NaBr or 3% KI; this allowed for the isolation of 12 new cytochalasins. Among them, two represented the first iodinated cytochalasins, while the brominated one displayed potent cytotoxicity against MDA-MB-435 cells derived from M14 melanoma cells [101]. Qin and colleagues identified nine new (DMOA)-derived meroterpenoids, i.e., peniciacetals A−I, through the fermentation of the mangrove-derived fungus *Penicillium* sp. HLLG-122 in different media and the subsequent MN analysis. Peniciacetals B, E, and F had good cytotoxicity against the MCF-7 cell line [100].

Following the bioactivity assessment of a microbial community isolated from the deep-sea sediments, Magot and colleagues selected the fungus *Aspergillus versicolor* PS108-62 for an in-depth chemical investigation. The metabolomic profile was evaluated in four different culture conditions that differed in carbon and nitrogen source, and salinity. Also here, the nutritional source affected the chemiodiversity of compounds and the antibiotic activity; in particular, rice was the best medium in terms of yield, while the addition of bromide unlocked the production of metabolites that inhibited the growth of *C. albicans*. In addition, subsequent chemical investigations of this strain led to the isolation of a new versicolide A, a new quinazoline (−)-isoversicomide A, and the well-known metabolites burnettramic acid A, cyclopenol, and cyclopenin. The evaluation of the bioactivity of the two new compounds was not evaluated due to the low yield obtained, while burnettramic acid A was found to be a potent inhibitor of *C. albicans* growth (IC_50_ 7.2 μg/mL) [104]. Moreover, the marine fungus *Alternaria alternata* LW37, isolated from deep waters, was cultivated in six different media, of which five were liquid and only one solid consisted of rice; the latter resulted in the most prolific medium, leading to the isolation of three new derivatives of dibenzo-α-pyrone, alternolides A–C, plus seven known compounds. The new compounds alternolides B and C exhibited inhibitory effects against α-glucosidase [135]. In the same way, a new secondary metabolite, 4-methoxy-3-[4-(acetyloxy)-3-methyl-2-butenyl] benzoic acid, along with 11 known compounds were isolated from the fermentation products of the marine-derived fungus *Phomopsis lithocarpus* FS508. The novel compound did not exhibit any activities in the cytotoxicity assay against the HepG-2 human liver cancer cell line or in the anti-inflammatory assay [105]. Zhao and collaborators isolated novel 3-decalinoyltetramic acid (3DTA), fusarisetins B, C, and D, and four known derivatives, including equisetin. The equisetin yield was optimized by culturing the marine-derived fungus *Fusarium equiseti* D39 strain in seven different media containing carrots, maize kernels, malt, tuberous roots of potatoes, peanuts, and soybeans. Moreover, different salt concentrations were used. The most favorable condition for producing equisetin was achieved using potato liquid medium with 1% salinity. As for bioactivities, all produced compounds displayed obvious phytotoxicity [97]. In addition, changing the media composition of the fermentation of the fungal strain *Emericellopsis maritima* BC17, isolated from sediments of the intertidal zone in Cadiz, four new eremophilane-type sesquiterpenes were isolated; however, they showed no cytotoxic or antimicrobial activities [136]. In 2021, Yao and colleagues applied an OSMAC regime to study the metabolomic profile of the marine fungus *Aspergillus* sp. SCSIO 41501 by altering culture medium compositions, resulting in the isolation of three new cyclohexenone derivatives, aspergispones A–C, and five new cyclohexenone derivatives, aspergispones D–H [99]. Moreover, different cultivations guided by the OSMAC strategy were applied to induce the production of metabolites in 21 fungal isolates from the Atlantic sponge *Grantia compressa*, allowing the choice of the promising fungus *Eurotium chevalieri* MUT 2316, as a producer of 10 compounds with promising antiviral and antibacterial activities [137].

Nevertheless, despite the large number of novel molecules identified by this method, implementing this approach can be time-consuming, and the challenge remains in identifying conditions that can effectively stimulate the synthesis of all the intriguing and beneficial compounds potentially produced by organisms.

#### 2.2.2. Co-Cultivation in Marine Microbial Drug Discovery

Updates on co-cultivation techniques applied to marine microbes, which have improved the diversity of the metabolic profile, and screening approaches for the rapid identification of novel metabolites were highlighted [73,138,139,140]. In recent years, advanced technologies like microfluidics, next-generation 3D bioprinting, and single-cell metabolomics have improved microbial communication in large-scale co-cultivation experiments [141]. One effective strategy includes the use of physical scaffolds, like cotton or talc, which were shown to enhance metabolite production in mixed fermentation [142]. Additionally, another established strategy involves the use of elicitors, such as stress induced by metal additions or inducers like N-acetyl-d-glucosamine (GlcNAc) and histone deacetylase inhibitors (HDAC). Additionally, omics techniques, such as genomics and proteomics, are relevant to rapidly assessing the effectiveness of different consortia, as highlighted by Rasheed et al. [143].

This paragraph will consider studies conducted in the last 10 years focused on new marine molecules identified by co-cultivation, highlighting only the compounds not included in previously published reviews (Table 1).

For example, co-cultivating *Streptomyces* sp. GET02.ST and *Achromobacter* sp. GET02.AC isolated together from the gut of the wharf roach, *Ligia exotica*, in Korea’s intertidal zone, induce the production of two new metabolites, ligiamycins A and B, which were significantly increased compared with the pure culture. Both ligiamycin A and ligiamycin B displayed antibacterial effects against *S. aureus*, while ligiamycin B exhibited moderate cell cytotoxicity against human colorectal carcinoma cell line (HCT116) [90]. Similarly, a co-culture of the two bacteria *Janthinobacterium* spp. ZZ145 and ZZ148, isolated from the same marine sediment sample, produced distinctive secondary metabolites with stronger bioactivity than axenic cultures. In particular, two new polyketides, janthinopolyenemycins A and B, were isolated only in the co-culture, and both showed antifungal activity against *C. albicans* [89]. This technique successfully induced the expression of silent gene clusters through elusive crosstalk mechanisms between species [68]. Diffusion of signal molecules promoted the biosynthesis of two new cytotoxic and antifungal polycyclic tetramate macrolactams, i.e., umezawamides A and B, from a combined culture of *Umezawaea* sp. RD066910 and the mycolic acid producer *Tsukamurella pulmonis* TP-B0596 [144].

Fungi are the microorganisms with the highest discovery rate of new compounds using the co-culture approach [140]. An interesting approach was used by Oppong-Danquah et al. who co-cultivated the marine fungus *Cosmospora* sp. with the phytopathogen *Magnaporthe oryzae*, which was used as a model competitor to activate the transcription of cryptic BGCs. In fact, five isochromanones with anti-phytopathogenic activities, including the two new soudanones H-I, were isolated in the co-culture, but were not produced in the axenic fungal cultures [107]. Beyond resource limitations, physical contact during co-cultivation induces stress on the fungal cell wall, resulting in changes in the production of compounds that serve as second messengers or act directly [145]. Yurchenko and collaborators analyzed the variation in the metabolic profile of *Aspergillus fumigatus* KMM 4631 culture and its co-cultures with other marine fungi. Although the profiles of the monocultures were similar to those of co-cultures, the joint cultivation with *Penicillium hispanicum* KMM 4689 led to the identification of two unidentified novel compounds, enhancing the DPPH radical scavenging and urease inhibition activities in the co-culture, a signal of activation of the defense system by one of the two microorganisms in the culture [108].

In the case of the fungus *Beauveria felina* KMM4639 isolated from marine sediments collected at a depth of 10 m (Van Phong Bay, South China Sea, Vietnam), co-cultivation with *Aspergillus carneus* KMM4638 led to the identification of three new drimane-type sesquiterpenes, asperflavinoids B, D, and E, some of which had moderate cytotoxic activity against MCF-7 cells [103]. In a subsequent study by Belousova, the marine fungus KMM4639 was subjected to further in-depth molecular analyses, re-identified in genus *Amphichorda*, and a new chemical investigation, based on a mixed fermentation with *A. carneus* KMM4638. This approach resulted in the discovery of five new quinazolinone alkaloids, named felicarnezolines A–E, along with a novel highly oxygenated chromene derivative oxirapentyn M, and five previously reported related compounds. The novel compound felicarnezoline B demonstrated a high cytoprotective potential, increasing the viability of CoCl2-treated SH-SY5Y (human neuroblastoma cell line) and H9c2 (a subclone of the original clonal cell line derived from embryonic BD1X rat heart tissue) of 72.6% and 19.1%, respectively [109].

Also for microalgae, mixed fermentation with other microbial species or other microalgae has proven to be very successful in increasing the production of pigments and biomass [86,146]. Moreover, these consortia were explored not only for metabolite production but also for applications in waste remediation, carbon capture, biofuel production, and other environmental sustainability initiatives [147,148,149,150,151]. A binary culture of two species of microalgae, *Phaeodactylum tricornutum* and *Aurantiochytrium limacinum*, represented a novel and alternative cultivation strategy for the development of omega-3 fatty acids for application as feed in aquaculture [152].

## 3. Drug Discovery in the Metabologenomics Era: Recent Advances and Perspectives

### 3.1. Metabologenomics: Basic Principles and Tools

Traditionally, the common approach used for NP isolation is based on the biological screening of extracts to identify metabolites responsible for biological properties, followed by bioassay-driven purification using chromatographic methods and structural characterization through mass spectrometry (MS) and nuclear magnetic resonance (NMR) spectroscopy [153]. However, this workflow shows many limitations, including high rediscovery rates of known NPs, a potential high ecological impact due to sample collection, and insufficient quantities of NPs for characterization [154].

Recently, significant advancements in technologies for genomic and metabolomic data acquisition, and bioinformatics tools for their analysis, are leading to a new wave in NP analyses. Therefore, over the past 10 years, the genome sequencing-driven approach has been introduced as an alternative to the traditional NP discovery process [155].

The genome mining strategy exploits genomic information to discover bioactive NPs. In detail, the genome mining technique allows for the analysis of BGCs, i.e., cluster of gene coding for enzymes responsible for the biosynthesis of a metabolite and its chemical variants [1]. Knowing the activity of the enzymes is useful to hypothetically predict the structure of new NPs [156,157]. This is particularly true in the case of non-ribosomal peptide synthetases (NRPSs) and polyketide synthases (PKSs), where there is a close collinearity between the modular architecture of biosynthetic systems and the chemical structures of the encoded metabolites [158]. At the same time, this approach uncovers BGCs exhibiting homology with genes involved in the production of known natural compounds, reducing the rediscovery rates [159]. Thus, despite the high structural variability of microbial secondary metabolites, biosynthetic machineries are often conserved [158].

A plethora of bioinformatic tools (Table 2) are available to perform genomic data analysis, like the Basic Local Alignment Search Tool (BLAST) [160], which compares query sequences with a database of already known sequences and selects similar sequences to the one of interest. Another example is the PRISM platform (PRediction Informatics for Secondary Metabolomes) [161,162], which allows for the detection of NRPSs, PKSs, terpenes, RiPP, glycosides, alkaloids, and phosphonates BGCs, and operates with a combinatorial approach for genome-driven chemical structure prediction of genetically encoded NRPs and type I and type II PKs. Nowadays, the most widely used bioinformatics software is AntiSMASH 7.0 (Antibiotics and Secondary Metabolites Analysis Shell), which is specialized in the detection of BGCs from bacterial, plant, or fungal genomes in chemical prediction and in the alignment of homologous sequences [163,164]. In addition, NAPDoS (Natural Product Domain Seeker) is used to identify biosynthetic enzymes. In particular, it can identify characteristic structures of PKs and NRPSs [165]. In 2015, the MIBiG (Minimum Information about BGCs) Data Standard and Repository was created to have a standardized data format for the scientific community [154]. The MIBiG repository reports minimal and unique information on BGCs, and selects all BGCs with known functions and secondary metabolites produced [166]. This database is very useful because it performs genomic dereplication, reducing the risk of discovery of known compounds by BGC sequence homology on a large dataset [155].

**Table 2:** List of bioinformatics tools used for genome mining. URLs have been accessed on 6 November 2024.

Genome Database	URLs
GenBank/NCBI	https://www.ncbi.nlm.nih.gov/
RefSeq	https://www.ncbi.nlm.nih.gov/refseq/
In-house genome database	https://ngdc.cncb.ac.cn/gwh/
**BGC identification**	
BLAST	https://blast.ncbi.nlm.nih.gov
antiSMASH	https://antismash.secondarymetabolites.org
FASTA	https://www.ebi.ac.uk/Tools/sss/fasta/
RIPper	https://github.com/TheRIPper-Fungi/TheRIPper
PRISM	https://prism.adapsyn.com/about
MetaBGC	https://github.com/donia-lab/MetaBGC
RODEO	https://bio.tools/rodeo
pHMMs	https://arxiv.org/abs/2207.09765
**BGC database**	
MiBIG	https://mibig.secondarymetabolites.org
IMG-ABC	https://img.jgi.doe.gov/cgi-bin/abc/main.cgi
BIG-FAM	https://bigfam.bioinformatics.nl/home
antiSMASH-DB	https://antismashdb.secondarymetabolites.org
ARTS-DB	https://arts-db.ziemertlab.com/
**BGC analysis**	
Big-SCAPE	https://github.com/medema-group/BiG-SCAPE
BLAST	https://blast.ncbi.nlm.nih.gov
EFI-EST	https://efi.igb.illinois.edu/efi-est/
ARTS	https://arts.ziemertlab.com
EvoMining	https://github.com/nselem/EvoMining/wiki
FastTree	https://bio.tools/fasttree#!

In addition, the methodological progress in modern analytical chemistry, especially in MS and NMR, enables the comprehensive analysis of natural extracts of increased complexity [167]. The most widely used technique for the analysis of complex extracts is high-performance liquid chromatography–tandem mass spectrometry (LC-MS/MS), which is an analytical technique that combines the multi-compound separation capabilities of high-performance liquid chromatography with the mass analysis capabilities of MS [168]. However, the huge number of data obtained from LC-MS/MS analysis makes manual analysis time-consuming. To simplify LC-MS/MS data inspection, efficient powerful data mining software were developed (Table 3), and one of them is molecular networking (MN) [169].

MN is an innovative bioinformatics strategy that facilitates and speeds up the analysis of LC-MS/MS data of complex extracts by providing a visual representation of the detected metabolites. Specifically, MN relies on the principle that metabolites with similar chemical architecture tend to generate similar MS/MS fragmentation spectra [170,171]. The major advantage of using this technique is its ability to compare the MS/MS spectra of compounds present within extracts with the fragmentation patterns of known NPs found in online libraries, i.e., Global Natural Product Social, GNPS (Table 3). This platform collects more than 200,000 MS/MS spectra of NPs, greatly facilitating the recognition of new compounds and avoiding the re-isolation of any known substances [172]. An approach called Feature-Based Molecular Networking, which consists of pre-processing the raw LC-MS/MS data using MS processing software like MZmine, was shown to generate significantly better networks [173]. Finally, the processed files are uploaded to the GNPS site, mapped, and visualized using the Cytoscape v.3.10.3 program [174]. GNPS also provides many annotation tools, facilitating the recognition of peptide and non-peptide compounds (i.e., Dereplicator and Dereplicator+) [175] and the chemical classification of compounds grouped in clusters (MolNetEnhancer workflow).

**Table 3:** List of bioinformatics tools used for MS-based molecular networking and natural product databases. URLs have been accessed on 6 November 2024.

MS Data Pre-Processing	URLs	Access Type
MzMine	https://mzmine.github.io/	Open Access
**MN generation**		
GNPS	https://gnps.ucsd.edu/ProteoSAFe/static/gnps-splash.jsp	Open Access
MetGem	https://metgem.github.io/	Open Access
**MN visualization**		
Cytoscape	https://cytoscape.org/	Open Access
**Natural Product database**		
GNPS	https://gnps.ucsd.edu/ProteoSAFe/static/gnps-splash.jsp	Open Access
Dictionary of Natural Products	https://dnp.chemnetbase.com/chemical/ChemicalSearch.xhtml?dswid=9675	Paid Access
Natural Product Atlas	https://www.npatlas.org/	Open Access
COCONUT	https://coconut.naturalproducts.net/	Open Access
MarinLit	https://marinlit.rsc.org/	Paid Access
Metlin	https://metlin.scripps.edu/landing_page.php?pgcontent=mainPage	Partially Paid

However, the above-mentioned tools are all dependent on LC-MS/MS spectral metabolomic data, which strongly rely on various steps of the metabolomic workflow (i.e., LC eluents, *m*/*z* window, ionization properties, and the quality of MS/MS spectra) [167]. Therefore, not the full metabolome of an organism, but only a subset of metabolites, is analyzed. Alongside MS, NMR spectroscopy has proven to be an essential tool in NP research. Although NMR is the mainstay for determining structures of unknown compounds, it was recently extended to the analysis of complex mixtures in the growing field of metabolomics [176]. Besides the standard ^1^H NMR spectrum and ^13^C NMR spectrum, homo- and hetero-nuclear 2D NMR experiments allow for a more efficient chemical shift assignment, providing information on the connectivity of atoms and spatial proximity within spin systems [177].

NMR spectroscopy gathers favorable properties, including its high reproducibility and robustness, its non-destructive, non-selective and non-invasive nature, and its ability to quantify metabolites. The analysis of complex mixtures by NMR has been extensively used to characterize natural extracts [177]. Although 2D NMR experiments provide more detailed information on a mixture and possess a superior resolution, they are characterized by longer acquisition times, which makes them less suitable for metabolomic studies. Therefore, ^1^H NMR is the most widely used nucleus in NMR-metabolomics [177]. The general workflow used for NMR-metabolomic studies involves the detection of metabolite signals, followed by careful database search and quantifying the identified metabolites using a single internal standard. Nowadays, the largest and freely available database housing NMR data for NPs is the Natural Product Magnetic Resonance Database (NP-MRD) [178].

It is worth mentioning Small Molecule Accurate Recognition Technology (SMART), a newly developed AI-based dereplication tool used to streamline NP dereplication and structural elucidation. In detail, experimental 2D HSQC NMR data of newly isolated compounds are processed with the SMART algorithm and embedded in cluster maps with previously characterized compounds, in which each node represents an HSQC spectrum. Nodes recognized as known compounds by SMART are labeled with compound names, if available, or eventually with the source organisms. Molecular structure similarity can be inferred based on the distance of the connected nodes in the cluster maps [179].

In this scenario, we aim to report on the recently introduced integrated strategy defined “metabologenomics”. Combining genome mining to identify potential BGCs of specific secondary metabolites with metabolomics, which provides structural information of expressed secondary metabolites through the evaluation of chemical profiles, metabologenomics has proven to be a valuable approach for potential drug discovery [154,180,181]. To this aim, some significant stories arising from the successful application of this hybrid approach will be discussed in detail in Section 3.2.

### 3.2. Marine Natural Products Discovered Through Metabologenomics

Different workflows for integrated approaches in metabologenomics were described by Avalon and collaborators [154]. An integrated strategy could start with the dereplication of BGCs through global databases. Thanks to the evaluation of the homology of BGC sequences involved in the biosynthesis of known NPs, it is possible to prioritize interesting BGCs for in-depth investigation, followed by metabolomic analysis. An alternative approach is based on an initial screening of microbial extracts via GNPS-molecular networks, identifying potential new chemistry followed by biosynthetic analysis to mine structural features.

**Figure 3:**
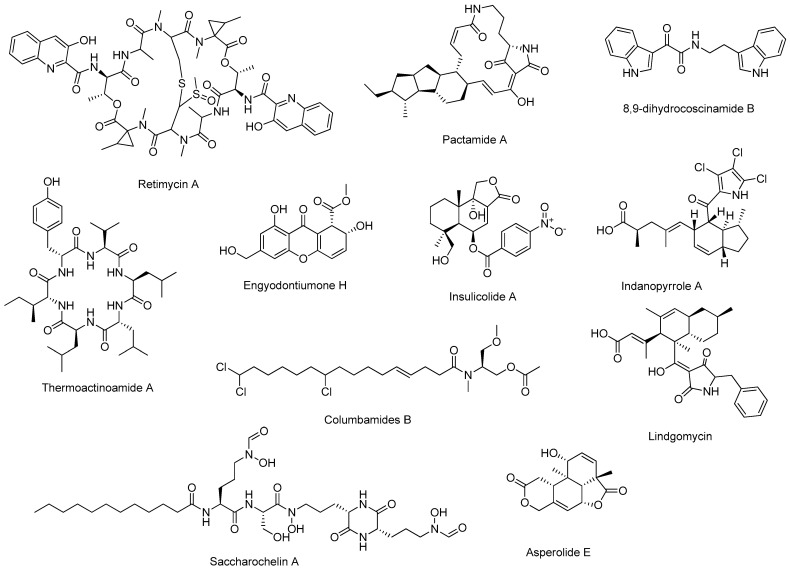
Chemical structure of most promising new NPs isolated through metabologenomic approach from marine bacteria, cyanobacteria, and fungi.

Examples of NPs discovered in the last ten years from marine bacteria, cyanobacteria, and fungi through a combined genomics and metabolomics approach are listed in Table 4. The chemical structure of the most promising novel NPs reported in the Table 4 are shown in Figure 3, and some interesting examples are discussed in this paragraph. However, this approach can also be enhanced by integrating innovative cultivation techniques to promote the synthesis of novel compounds (some examples were given in Section 2.2.1). Despite the potential of metabologenomics, its application to microalgae has not yet led to significant discoveries. This limitation is attributable to several challenges, including the high metabolic complexity of microalgae, characterized by dynamic biosynthetic networks that respond to diverse environmental stimuli. Such complexity complicates the identification of new metabolites. This is compounded by the poor genomic characterization of many microalgal species, which hinders the integration of genomic and metabolomic data that is essential for the success of this approach.

The usefulness of the two paired omics approach was demonstrated by identifying a new group of antibiotics, thiomarinols. Thirteen strains closely related by gene sequence similarity to *Pseudoalteromonas luteoviolacea* were analyzed in search of new secondary metabolites. Strains 2ta16 and NCIMB1944 were recognized to be responsible for producing novel NPs, thiomarinols, NRPS-PKS hybrid compounds based on pseudomonic acid and pyrrothine. The putative biosynthetic pathways were predicted by genome mining with antiSMASH 2.0. Following MS/MS analysis and MN, a group of thiomarinol and pseudomonic acid analogs were identified, including two unknown compounds, showing antibiotic activity [182].

The efficient discovery of new NPs by integrating imaging MS and MN was evidenced in the study of the marine bacterium *Vibrio* sp. QWI-06 extracts, leading to identifying a series of amino-polyketide derivatives, vitroprocines A–J. From an antibacterial screening of 265 marine-derived microbes, 19 bacteria exhibiting significant anti-microbial activities were selected. Thanks to a comparative metabolomic analysis of two *Vibrio* strains, a group of amino-polyketide, vitoprocines, was identified. The structures were defined by NMR and HR-MS/MS spectra. The structure–activity relationship of isolated vitroprocines suggested that metabolites A2, B1–B4 were active against *A. baumannii*, a pathogen involved in nosocomial infections, whereas the metabolites of subgroups C, D, and F were inactive [183]. Metabologenomics turned out to be a valuable approach to unlock the biosynthetic machinery of the novel Antarctic deep-sea isolate *Lacinutrix shetlandiensis* sp. nov. WUR7. Genomic investigation of this strain highlighted an unusual tryptophan decarboxylase, which was exploited to drive the bacterial metabolism toward indole-based alkaloid biosynthesis, thus leading to the chemoinformatic annotation of several β-carboline, bisindole, and monoindole alkaloids, alongside with the isolation of the antibacterial bisindole 8,9-dihydrocoscinamide B [92].

Another productive application of a metabolomic-based approach combined with genome mining enabled the successful isolation and characterization of a new bioactive NP, wenchangamide A, as well as the structural prediction of its analog wenchangamide B from an environmental collection of the marine filamentous cyanobacterium, cf. *Neolyngbya* sp. First, a broad overview of the chemical composition of the extract was obtained with MS-based MN, followed by the putative annotation of detected compounds. Wenchangamide A was then isolated and its structure fully elucidated, revealing a lipopeptide structure, whose biosynthesis follows the NRPS/PKS hybrid biosynthetic mechanism, already found in other cyanobacteria. Further studies on the molecular cluster containing wenchangamide A led to the identification of a new analog, wenchangamide B. Since its quantity was not enough for pure compound isolation, its structure was predicted thanks to MS/MS data and biosynthetic rationale. The wenchagamide family is an attractive class of compounds, thanks to the demonstrated activity of wenchangamide A to induce apoptosis in HCT116 human colorectal carcinoma cell line in vitro [184].

Another productive application of the metabolomic approach integrated with genomic analysis led to the discovery of columbamides, a new class of acylamides isolated from the marine cyanobacterium *Moorea bouillonii* PNG. An initial analysis of the genome of *M. bouillomii* helped in the detection of BGCs showing homology with published cyanobacterial genomes. The genome of *M. bouillonii* PNG showed seven PKS/NRPS-based biosynthetic pathways, including two clusters producing di- and tri-chlorinated compounds. Subsequently, the potential expression of new metabolites was evaluated by MN. The presence of an unannotated cluster of compounds, whose parent mass spectra showed the isotopic pattern of di- and trichlorinated species, suggested the presence of unknown NPs. Three new compounds, coloumbamide A-C, were isolated and their chemical structures elucidated by NMR. The most abundant isolated compounds were evaluated for their cannabinomimetic properties, inhibiting CB_1_ and CB_2_ receptors [185].

Among fungal metabolites uncovered by combining genomics and metabolomics, it is worth mentioning the two new series of 15-res peptaibols, named endophytins. *Trichoderma endophyticum* MMSRG85 is recognized as a producer of peptaibols, peptides consisting of chains of 5 to 20 amino acid residues, synthesized by NRPS, and showing remarkable biological properties, such as antimicrobial and antitumor activities [186]. Genome analysis identified 53 BGCs annotated with the FungiSMASH platform. Two NRP-synthetase biosynthetic machineries were considered potentially suitable for peptaibol production. The peptaibols characterized were divided into two groups: endophytins A1–A13 and endophytins B1–B8. The actual production of hypomuricin, harzianins, and endophytins was confirmed after the analysis of MS/MS spectra and from the generated molecular network [186].

The metabologenomic approach was used in the study of the biosynthetic potential of the fungus *Rhodotorula mucilaginosa* 50-3-19/20B collected from the mid-Atlantic ridge. The different bioactivity (e.g., antiproliferative and antimicrobial activities) observed in two different culture media suggested a thorough analysis of the extracts through bioactivity-based MN. Following the antiproliferative activity, one known and four new glycolipids, defined as polyol esters of fatty acid (PEFA) derivatives, were isolated and structurally characterized. Interestingly, the above-mentioned bioactivities were lost after the isolation of the compounds in their pure forms. PEFA glycolipids showed differential degrees of acetylation and were proposed as biosurfactants. Illumina-based genome sequencing was used to perform BGCs analyses, finding key components of the biosynthesis of PEFA [187].

The integration of NMR-metabolomic and genomic datasets was recently introduced in the discovery of new NPs, allowing for a better identification of structural properties as well as of key biosynthetic information [188]. Recently, NMR-metabolomic profiling and genome mining approaches led to the identification of new cyclic decapeptides from the extracts of the marine-derived *Streptomyces* sp. S063. First, ^1^H NMR spectrum of the culture extract of *Streptomyces* sp. S063 was recorded with the aim of fully analyze the bacterial metabolome. The interesting observation of unusually detected NMR chemical shifts in the negative chemical shift region suggested the presence of unknown NPs. The bioinformatic analysis of the genome shed light on the presence of an NRPS gene cluster (*len*) including piperazic acid-encoding genes. The combined information allowed the isolation and full structural elucidation of the cytotoxic piperazic acid-containing peptides, lenziamide D and B1 [188].

The demonstrated efficacy of the integrated approach of genomics and metabolomics is driving the development of innovative tools for collecting paired datasets, coupling genomic information to molecular spectra. In this regard, free web platforms and bioinformatic tools were designed to facilitate the complementary analysis of genomic and metabolomic data and, consequently, link gene clusters to their products. These computational techniques simultaneously compare and process data from thousands of organisms through different algorithms, starting with hypothesizing products encoded by BGCs, predicting the fragmentation spectra of the hypothetical molecules, and comparing the experimental spectra with the theoretical ones with the following computation of the statistical significance of the matches; examples of these techniques are NRPquest for NRPs [189], and MetaMiner, and DeepRiPP for RiPPs [190,191]. A comprehensive and updated platform called Paired omics Data Platform (https://pairedomicsdata.bioinformatics.nl/ accessed on 6 November 2024) linking genomic and metabolomic data was recently developed to provide a centralized database for paired datasets to further the discovery of new natural entities [167]. The results lead to (i) integrated and combined omics data analysis of metabolites and their BGCs, (ii) a reduction in dereplication times, i.e., identification of new compounds by structural similarities with known metabolites, and (iii) structural and bioactivity predictions of NPs [167].

**Table 4:** List of new NPs isolated through metabologenomic approach from marine bacteria, cyanobacteria, and fungi.

Microorganism	Origin	Place	Class	Compound	Bioactivity	Test	Activity Value	References
**BACTERIA**								
*Saccharomonospora* sp. CNQ-490	Marine sediment	Submarine Canyon, California, USA	NRPS	Taromycin A	Antibacterial	Microdilution broth	MIC ranging from 12.5 to 50.0 μg/mL vs. different strains of methicillin-resistant *S. aureus* (MRSA) and vancomycin-resistant *E. faecium* (VRE)	[192]
*Salinispora arenicola* CNS-205	Deep-sea sediments	Palau	Terpenoids/isoprenoids	Isopimara-8,15-diene-19-ol	Not reported	Not reported	Not reported	[193]
*Vibrio harveyi*	Sponge (*Tectitethya crypta*)	Long Key/Big Pine, Florida, USA	Nucleoside	Spongosine	Anti-inflammatory	NO production inhibition	3 µg/mL	[194]
					Analgesic vasodilation	Not reported	Not reported	
*Salinispora arenicola* CNT-005	Anellida	Fiji	NRPS	Retimycin A	Antiproliferative	Not reported	IC_50_ <0.076 μg/mL on HCT116	[195]
*Salinispora tropica* CNB-440	Marine sediment	Bahamas	Terpenoids/isoprenoids	Sioxanthin	Not reported	Not reported	Not reported	[196]
* Vibrio * sp. QWI-06	Marine sediment	Pingtung County, Taiwan	PKS	Vitroprocines A–J	Antibacterial (Vitroprocines A2, B1, B4)	Microdilution broth	MIC ≤ 8, 32, and 16 μg/mL against *A. baumannii* ATCC 19606L	[183]
*Salinispora pacifica* CNS-237	Deep-sea sediments	Palau	PKS type I	18-dihydro-14-hydroxyrosamicin, 18-dihydro-21-hydroxyrosamicin, 14-hydroxyrosamicin	Antibacterial	Not reported	MIC 50–100 μg/mL vs. *A. baumannii* ATCC17978 and *E. coli* CFT073 MIC 25–100 μg/mL vs. *P. aeruginosa* PA01 MIC 6.125–100 μg/mL vs. *S. pyogenes* NZ131 1.56–25 μg/mL vs. *S. aureus* USA300	[197,198]
*Pseudoalteromonas luteoviolacea* Strain 2ta16	Coral (*M. annularis*)	Florida Keys, Florida, USA	NRPS-PKS hybrid	Thiomarinol	Not reported	Not reported	Not reported	[182]
*Pseudoalteromonas luteoviolacea* NCIMB1944	Surface water	Nice, France, Mediterranean						
*Streptomyces* sp. JAMM992	Deep-sea sediment	Kinko Bay, Kagoshima, Japan	NRPS	Surugamide F	Antiproteolytic	Cathepsin B inhibition	No activity	[199]
*Streptosporangium* sp. CGMCC 4.7309	Marine sediment	Lijiao Bay, Huanghai Sea, China	PKS Type II	Hexaricins A–H	Antioxidant (A,C–H)	DPPH∙ Scavenging	EC_50_ < 45 μM (E-F < 3 μM)	[200,201]
						OH^−^ Scavenging	EC_50_ < 1530 μM (E-F < 620 μM)	
						O_2_^−^ Scavenging	EC_50_ < 1110 μM (E-F < 140 μM)	
*Streptomyces pactum* SCSIO 02999	Deep-sea sediment	South China Sea	PKS–NRPS hybrid	Totopotensamide TPMC	Antibacterial	Microdilution broth	No activity	[202]
*Streptomyces* sp. MP131-18	Deep-sea sediment	Trondheim fjord, Norway	PKS	Lagunapyrone D and E	Antibacterial	Disk diffusion	No activity	[203]
*Marinactinospora thermotolerans* SCSIO 00652	Deep-sea sediment	South China Sea	RiPPs	Mathermycin	Antibacterial	Agar diffusion	1 μM vs. *B. subtilis* LH45	[204]
* Streptomyces * sp. PKU-MA00045	Sponge (*Sinularia* sp.)	Not Reported	PKS type II	Fluostatins M–Q	Antibacterial; anti-inflammatory; cytotoxic	Not reported; NO inhibition; not reported	No activity	[205]
*Fulvivirga sp. W222*	Marine sediment	Aoshan Bay, Qingdao, China	*NIS*	*Fulvivirgamides A2*, *B2*, *B3*, *and B4*	Cytotoxic	MTT	A2, B2, B3 IC_50_ from 9 to 19 μM on A549, SMMC-7721 and HCT-116	[206]
*Streptomyces pactum* SCSIO 02999	Deep-sea sediment	South China Sea	Hybrid PKS–NRPS	Pactamides A–F	Cytotoxic	SRB	IC_50_ from 0.5 to 26 μM on SF-268, MCF-7, NCI-H460, HepG2	[207]
* Thermoactinomyces vulgaris *	Coastal hydrothermal vents	Iceland	NRPS	Thermoactinoamides A–K	Antiproliferative (A)	Real-time proliferation monitoring	5 μM on BxPC-3	[208]
					Antibacterial (A)	Microdilution broth	MIC 35 μM vs. *S. aureus* ATCC 6538	
*Saccharothrix* sp. D09	Intertidal zone sediment	Fushan Bay, Qingdao, China	NRPS	Saccharochelins A–H	Cytotoxic	MTT	IC_50_ from 1 to 20 μM on A549, MCF-7, HepG2, and HCT-116	[64]
* Lacinutrix shetlandiensis * sp. nov. WUR7	Deep-sea sediment	South Shetland Trough, Antarctica	Tryptophan decarboxylase	Indole alkaloids	Antibacterial (8,9-dihydrocoscinamide B)	Microdilution broth	IC_50_ 14.0 µg/mL vs. *S. aureus* and 39.1 µg/mL vs. MRSA	[92]
*Streptomyces* sp. S063	Marine sediment	Xinghai Bay, China	NRPS	Lenziamide D and B1	Cytotoxic	MTT	IC_50_ from 8 to 24 μM on HEL, H1975, H1299, A549–taxol.	[188]
*Streptomyces* sp. CNY-716	Marine sediment	Not reported	PKS	Indanopyrrole A and B	Antibacterial	Microdilution broth	(A) MIC 4 μg/mL, 2 μg/mL, 4 μg/mL, 2 μg/mL 4 μg/mL and 1–2 μg/mL against *E. coli* lptD4213, MRSA TCH1516, *Streptococcus* M1T1, VR-*Enterococcus faecium* DAPS, MR-*Staphylococcus epidermis* and *Haemophilus influenzae*, respectively	[209]
					Cytotoxic	MTT	after 24 h at 16 μg/mL vs. A549 indicating an antibiotic therapeutic index of 4–8	
**CYANOBACTERIA**								
* Moorea bouillonii * PNG	Coral (*Stylophora pistillata*)	Pigeon Island, Papua New Guinea	NRPS/PKS hybrid	Columbamides A Columbamides B Columbamides C	Cannabinomimetic	Displacements	Potent ligands of cannabinoid receptors CB_1_ and CB_2_	[185]
* Moorea producens JHB *	Shallow water	Hector’s Bay, Jamaica	NRPS	Hectoramide	Not reported	Not reported	Not reported	[210]
*Neolyngbya* sp	Intertidal zone	Bangtang Bay, Hainan, China	NRPS/PKS hybrid	Wenchangamide A Wenchangamide B	Antiproliferative	XTT	60% of cell viability reduction at 25 µg/mL on HCT116	[184]
**FUNGI**								
*Engyodontium album* DFFSCS021	Marine sediment	South China Sea	PKS	Engyodontiumones A–J 2-methoxyl cordyol C	Cytotoxic; antibacterial (Engyodontiumones H)	MTT	IC_50_ 4.9 μM on U937	[211]
						Microdilution broth	MIC ≤ 64 μg/mL vs. *E. coli* and *B. subtilis*	
*Aspergillus* sp. 16-02-1	Deep-sea sediment	Lau Basin hydrothermal vent, Southwest Pacific	PKS	Aspiketolactonol Aspilactonols A–F Aspyronol Epiaspinonediol	Cytotoxic (Aspyronol, Epiaspinonediol)	MTT	Aspyronol, IC_50_ of 241.2 μM on HL-60 Epiaspinonediol, IC_50_ 260.6 μM on K562 and 192.9 μM on HL-60	[212]
*Aspergillus ochraceus* Jcma1F1	Marine alga (*Coelarthrum* sp.)	Paracel Islands, South China Sea	Terpenoid	6b,9a-dihydroxy-14-*p*-nitrobenzoylcinnamolide (1) Insulicolide A	Antiviral	Cytopathic effect protection	IC_50_ 17.0 μM vs. H3N2 and 9.4 μM vs. EV71	[213]
					Cytotoxic	CCK8	IC_50_ ranging from 1.95 μM to 6.35 μM against 10 human cancer lines	
*Lindgomycetaceae* KF970 *Lindgomycetaceae* LF327	-	Arctic	PKS	Lindgomycin Ascosetin	Antimicrobial	Microdilution broth	IC_50_ ranging from 2.0 to 18.0 µM vs. *B. subtilis* DSM 347, *X. campestris* DSM 2405, *S. epidermidis* DSM 20044, *S. aureus* DSM 18827, MRSA, *C. albicans* DSM 1386, *S. tritici*, *P. acnes* DSM 1897	[214] [215]
	Sponge (*Halichondria panicea*)	Kiel Fjord, Baltic Sea, Germany						
*Penicillium* sp. Y-50-10	Hydrothermal vent sediment	Kueishantao, Taiwan	PKS	Methyl-isoverrucosidinol	Antimicrobial	Microdilution broth	MIC 32 μg/mL vs. *B. subtilis* CMCC (B) 6350	[216]
*Aspergillus wentii* SD-310	Deep-sea sediment	South China Sea	Terpenoid	Aspewentin A and D–M	Antimicrobial (I-M)	Microdilution broth	MIC ranging from 4 to 32 μg/mL vs. *E. coli* QDIO-1, *A. hydrophilia* QDIO-3, *E. tarda* QDIO-4, *P. aeruginosa* QDIO-6, *V. anguillarum* QDIO-8, *V. harveyi* QDIO-9, *V. parahaemolyticus* QDIO-10, *F. graminearum* QDIO-13	[217,218,219]
				Asperethers A–E	Cytotoxic	MTT	IC_50_ ranging from 10 to 48 μM on A549, HEK293, MCF-7, SMMC-7721, and T-47D	
				Asperolides D and E	Antibacterial; cytotoxic (E)	Microdilution broth, MTT	MIC 16 µg/mL vs. *E. tarda* IC_50_ ranging from 10.0 to 16.0 µM on HeLa, MCF-7, and NCI-H446	
*Stachybotrys longispora* FG216	Not reported	Not reported	Hybrid PKS–NRPS	FGFC4–7	Fibrinolytic activities (FGFC6-7)	Nitroaniline formation	25 µg/mL	[220]
*Penicillium granulatum* MCCC 3A00475	Deep-sea sediment	Prydz Bay, Antarctica	Terpenoid	Spirograterpene A Conidiogenone C and I	Antiallergic (Spirograterpene A)	Antiallergy assay	18% inhibition on E (IgE)-mediated rat mast RBL-2H3 at 20 µg/mL	[221]
*Trichoderma* sp. FM652	Marine sediment	Hanauma bay, Hawaii, USA	PKS	Sorbicillinoid 1–2	Antiproliferative (Sorbicillinoid 1)	Inhibition of NF-κB	IC_50_ 13.83 µM	[222]
*Trichoderma endophyticum* MMSRG85	Ascidian (*Botrylloides giganteus*)	São Paulo, Brazil	PKS-NRPS hybrid	Endophytins A1–A13 Endophytins B1–B8 Hypomuricina Harzianin HC	Not reported	Not reported	Not reported	[186]
*Beauveria felina* SYSU-MS7908	Ascidian	Xisha Islands, South China Sea	NRPS	Isaridin I-N	Antifungal (Isaridin J)	Agar dilution	EC_50_ 50 µg/mL vs. *G. citriaurantii*	[223]
*Exophiala xenobiotica* SDU 039	Deep-sea sediment	Not reported	HR-PKS and NR-PKS	Exopxenmycins A–G	Not reported	Not reported	Not reported	[224]

PKS: polyketide synthase; NRPS: non-ribosomal peptide; NO: Nitric Oxide; DPPH∙: 2,2-difenil-1-picrylidrazyl radical; OH−: Hydroxyl radical; O2−: superoxide anion; XTT: cell proliferation assay; MTT: 3-(4,5-Dimethyl-2-thiazolyl)-2,5-diphenyl-2H-tetrazolium Bromide; Methoxynitrosulfophenyl-tetrazolium carboxanilide; SRB: Sulforhodamine B; NF-κB: Nuclear factor kappa-light-chain-enhancer of activated B cells; MIC: Minimum Inhibitory Concentration; IC50: half maximal inhibitory concentration; EC50: half maximal effective concentration; CCK8: Cell Counting Kit-8.

## 4. High-Throughput Screening Approach in Marine Drug Discovery

High-throughput screening (HTS) is an approach used in drug discovery that has gained popularity over the past three decades in industrial and academic research. It can be applied to chemical, genomic, protein, and peptide libraries, where the goal is the optimization of the drug discovery pipeline by screening large libraries against different biological targets at a high rate (e.g., a few thousand compounds per day or week) and extremely low cost. Thanks to the miniaturization of the systems, it is possible to test samples with only small samples to examine. Moreover, by implementing the automation and robotics of the whole process, there is the possibility of screening up to 100,000 compounds per day. HTS is particularly used in the pharmaceutical industry, as it is useful for characterizing metabolic, pharmacokinetic, and toxicological data on new drugs, reducing costs for their development [225]. For implementing the HTS approach, the use of multi-well microplates has been crucial, with the initial use of 96-well plates later replaced by higher-density microplates of up to 1586 wells. Besides the ability to test many compounds in a short period, the additional advantage of this method is the use of minimal total volumes of reagents (approximately 2.5–10 μL), resulting in reduced costs [226]. Ultra-high density plates (3456-well microplates), which allow for only 1–2 μL of total volume to be used, were also tried, but several technical obstacles were noted [227].

Generally, detection systems used for HTS are fluorescence, scintillation proximity assays, and luminescence, which facilitate analysis procedures and are highly sensitive. To date, most HTS were tested on microorganisms isolated from soil. For example, Laubscher and Rautenbach recently developed a simple and rapid preliminary screening method, the bioluminescent simultaneous antagonism assay (BSLA), used to identify unknown antimicrobial-producing bacteria from natural sources. BSLA allowed for the screening of 264 soil isolates, identifying 10 antibacterial-producing isolates and reducing isolates for downstream analysis by 96% [228]. Moreover, biofilm formation is among the most important factors that contribute to persistence and antibiotic resistance of pathogenic bacterial species, including *Vibrio cholera* [229] and *Pseudomonas aeruginosa* [230]. Thanks to the advent of new technologies in high-throughput high-fluorescence imaging and software for image visualization and analysis, Peach and collaborators for the first time used an image-based biofilm analysis in *V. cholerae* expressing GFP in a high-throughput 384-well format to identify new biofilm inhibitors by screening a library of 3080 small molecules [231]. This epifluorescence-based method for the quantification of biofilm inhibition proved to be more reproducible compared to traditional crystal violet tests, and allowed for the identification of new classes of biofilm inhibitors, including mefloquine analogs [231].

Subsequently, Navarro and colleagues used a similar approach to study *P. aeruginosa* biofilm inhibitors and dispersal agents. They generated 312 fractions from marine microorganisms that were analyzed through a high-throughput 384-well image-based screening platform for both biofilm inhibition and dispersal. Interestingly, to distinguish between classical antibiotics and non-antibiotic biofilm inhibitors/dispersants, complementary tests were developed to be performed in the same screening plate to simultaneously analyze both the metabolic activity of bacterial cells and the biofilm coverage. As a result of this study, the natural cyclic depsipeptide products skyllamycin B and C were identified as promising non-antibiotic biofilm inhibitor/dispersant agents. Skyllamycin B exhibited activity combined with azithromycin, suggesting that the simultaneous use of the two drugs can remove the biofilm covering and inhibit cellular activity [232].

Moreover, a new fast miniaturized screening method was recently developed to cultivate (in deep-well plates) and evaluate about 300 marine bacteria simultaneously to discover new antimicrobials and biosurfactants with membrane-disrupting activity. This method allowed for the selection of the deep-sea bacterium *Bacillus halotolerant* BCP32, collected from Dohrn Canyon (Gulf of Naples). This bacterium proved to be an excellent producer of surfactants and nobilamides. Among the main compounds, two novel nobilamides (T1 and S1) were identified, and S1 showed strong antibiotic activity against different Gram-positive pathogenic strains [233].

Conventional screening of microorganisms producing a carotenoid of interest is generally time-consuming and laborious, due to the high number of carotenoids present in nature that vary in structure and colors. For this reason, a 96-well plate-based HTS approach was developed, enabling the screening of a large number of colonies in a short time [232,234]. Thanks to this method, a library of 701 pigmented marine microbial strains, including bacteria and red yeasts, was used to isolate novel high-value carotenoids producers, demonstrating a high diversity of both carotenoids (e.g., astaxanthin, zeaxanthin, lutein, and canthaxanthin) and carotenoid-producing microbial strains. Moreover, some strains showed high selectivity for carotenoid production, and new producers of zeaxanthin and lutein were identified [234].

A novel method for screening antibacterial molecules was developed by modifying a 96-well plate with eight-cut wells. These cut wells allowed for the simultaneous testing of antibacterial activities of eight different marine fungal strains isolated from the hydrothermal vent digestive gland crab and sediment. Briefly, eight wells were cut, filled with agar medium that penetrated through the holes, and the different fungi were grown in each well to allow the molecules to diffuse into the agar. The antibacterial activity was evaluated against *Bacillus subtilis*, *S. aureus*, and *E. coli*, inoculated at the back of the wells. The most promising fungus, *Penicillium* sp. Y-5-2, was selected and cultivated on a large scale, and five new molecules named austinone, penicillisocoumarin A, penicillisocoumarin B, penicillisocoumarin C, and aspergillumarins A were identified [235].

The redox indicator resazurin was used for the development of a new fluorescence-based HTS of anti-Vibrio agents, which proved to be easy to use, sensitive, economical, and reliable [236]. For this purpose, this method was used to screen 1516 methanolic fractions obtained from 32 different marine fungal cultures. Four of these fractions extracted from four different strains (*Aspergillus flavus* MCCC 3A00246, *Anacystis nidulans* MCCC 3A00036, *Schizophyllum commune* MCCC Z16, and *Penicillium solitum* MCCC 3A00215) showed high inhibitory activity (>90%) against all tasted pathogenic strains of *Vibrio*. Moreover, an in vivo trial demonstrated that the fraction Z16-17, isolated from *S. commune* MCCCZ16, when used as a feed additive, reduces *Vibrio* infections in shrimp aquaculture [236]. A recent study [237] developed a new, miniaturized, and automated HTS platform for drug discovery in regenerative medicine to find compounds involved in the osteogenic or chondrogenic differentiation of human mesenchymal stem/stromal cells (hMSCs). In particular, a small library of molecules isolated from the marine fungus *Penicillium antarcticum*, collected from the tunicate *Aplidium pallidum*, was investigated. The new high-throughput osteogenic assay involved the detection of quantities of missing calcium in the culture medium (indirect method) compared to the classic method of quantifying mineralized calcium (direct method). For the chondrogenic test, the process was optimized with high-density monolayer cultures, eliminating the centrifugation phase of the classic method. The results showed that two new itaconic esters extracted from *P. antarcticum*, identified as ethyl 8-hydroxyhexylitaconate and ethyl 9-hydroxyhexylitaconate, acted as inhibitors of both osteogenic and chondrogenic differentiation of hMSCs. These compounds also showed anti-inflammatory activity, which is important for the treatment of diseases such as vascular calcification. Moreover, a new itaconic ester (methyl 8-hydroxyhexylitaconate) and two known hexylitaconic acid derivatives (methyl hexylitaconate and 9-hydroxyhexylitaconate) also showed a significant inhibition of hMSC osteogenic differentiation, at both 1 and 10 M. The assays were optimized with little plate manipulation to reduce variability to a minimum, in a 96-well plate format; however, switching to the 384-well plate format is feasible to enable the screening of large libraries of products.

Regarding microalgae, a high-throughput staining method for the detection of neutral lipids for biodiesel production was used in two different microalgae, *Chlorella ellipsoidea* and *Chlorococcum infusionum* [238]. The microalgae were grown and, subsequently, the staining efficiency was evaluated using different concentrations of Nile red and DMSO, in the presence of different physical and chemical parameters, including incubation period, temperature, and pH. In this study, this method was coupled with an FACS Verse flow cytometer to detect the accumulation of intracellular lipids, and with confocal microscopy to quantify their distribution in microalgal cells. Similarly, Nile Red fluorescence staining procedure, enhanced with either glycerol or DMSO, was used for the evaluation of neutral lipid in *Nannochloropsis* sp. [239]. Sudfeld and collaborators [240] developed a universal single-cell HTS protocol for lipid staining; they used *Nannochloropsis oceanica* to optimize the process of identification of lipids by the green fluorescent derivative BODIPY 505/515, demonstrating that stressed cells require a higher content of the fluorescent derivative for complete staining.

An HTS approach was used to identify new microalgae producers of high-value carotenoids, leading to the isolation of a new green marine microalga, *Auxenochlorella* sp. LEU27. Different growth parameters as temperature, pH, NaCl concentration, and time, were also used to optimize lutein production. For screening, the culture was grown using 96-well deep plates, and the extraction was performed using DMSO-methanol. The authors demonstrated the production of a large amount of carotenoids (~1203.9 ± 98 μg g^−1^ dry cells) of which lutein represented approximately 82.8% [241]. To improve the performance of diatoms in carotenoid production, *P. tricornutum* was used as a model to optimize an HTS method to monitor the carotenoid accumulation by chemical mutagenesis, carried out using ethyl methanesulfonate (EMS) and N-methyl-N′-nitro-N-nitrosoguanidine (NTG). Mutants with higher than 15% carotenoid content compared to wild-type strains were selected. Thanks to this HTS approach, starting from 1000 isolated strains, only five mutants were selected. Four of the five mutant strains were stable in the long term, and the best mutant showed a 69.3% increase in fucoxanthin production compared to the wild-type [242]. Phenotyping methods are often used to select strains for specific purposes, including the production of bioactive products. For this reason, Argyle and collaborators [243], based on a previous study [244], developed a high-throughput methodology for quantitative phenotypic assay of microalgae (QPA), where integrated phenotypic traits can be characterized simultaneously. Thanks to this new approach, out of 432 inoculated culture wells, 364 were characterized, leading to a new possibility of phenotyping fresh isolates. The authors showed that traits have a similar pattern if grown in small volume compared to large volumes, suggesting that this approach could also have potential applications in drug discovery [243].

**Figure 4:**
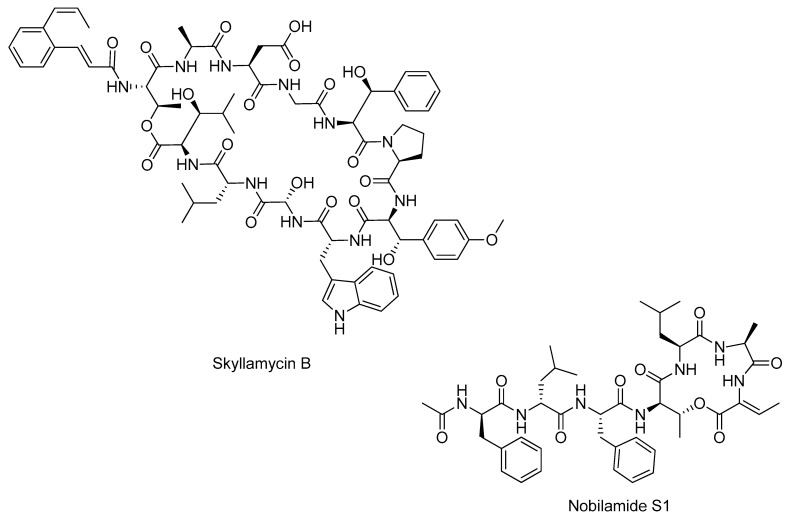
Chemical structure of most promising new natural products isolated from marine-derived bacteria and fungi by high-throughput screening approach.

Table 5 lists the novel compounds isolated from marine microorganisms and identified in the last 10 years through an HTS approach, and the chemical structure of the most promising novel NPs listed in the table are shown in Figure 4.

**Table 5:** List of new natural products isolated from marine-derived bacteria and fungi by high-throughput screening approach.

Microorganism	Origin	Place	Class	Compounds	Bioactivity	Assay	Activity Value	References
**BACTERIA**								
*Streptomyces* sp. 1675	Marine sediment	Westport Jetty, WA, USA	Depsipeptides	Skyllamycins B–C	Biofilm inhibitor and dispersant	Biofilm inhibition model biofilm dispersion model	*P. aeruginosa* biofilm inhibition EC_50_ 30 μM (B) and 60 μM (C)	[232]
*Bacillus* sp. BCP32	Marine sediment	Dohrn Canyon, Gulf of Naples, Italy	Depsipeptides	Nobilamides T1–S1	Antimicrobial (T1)	Microdilution broth	No activity	[233]
					Antimicrobial (S1)		MIC 15.6 μg/mL vs. *S. aureus* 6538p, 7.8 μg/mL vs. *S. aureus* 6538, 31.5 μg/mL vs. *S. xilosus* MB5209, 3.9 μg/mL vs. *Listeria monocytogenes* 677	
**FUNGI**								
*Penicillium antarcticum*	Tunicate (*Aplidium pallidum*)	Corranroo Bay, Galway, Ireland	Itaconate derivatives	ethyl 8-hydroxyhexylitaconate, ethyl 9-hydroxyhexylitaconate, methyl 8-hydroxyhexylitaconate methyl hexylitaconate, 9-hydroxyhexylitaconate	Osteogenic and chondrogenic differentiation inhibition	Osteogenic and chondrogenic assays	Inhibition of hMSCs from 1 to 10 µM	[237]
*Penicillium* sp. Y-5-2	Hydrothermal vent sediment	Kueishantao Island, Taiwan	Isocumarins Austin derivatives	Austinone Penicillisocoumarin A–C Aspergillumarins A	Antimicrobial	Combined microdilution broth and agar diffusion assays	MIC 32 μg/mL vs. *E. coli*	[235]

MIC: Minimum Inhibitor Concentration.

## 5. Metabolic Engineering

Metabolic engineering refers to targeted alterations of the metabolic pathways of an organism, performed to better understand these pathways and exploit this information to make it fruitful in terms of yield and productivity of a product of interest [245]. Metabolic engineering is a powerful approach utilized to optimize industrial fermentation processes by adding foreign genetic material using recombinant DNA technologies [246]. A main requirement is a deep knowledge of the cellular functions and organization of metabolic pathways. Furthermore, the possibility of performing metabolic engineering strategies goes along with the availability of proper molecular biology tools like strains and vectors capable of enabling rapid transformation with a good yield as well as the availability of lab strains.

In recent years, advances in gene editing tool kits, like zinc-finger nucleases (ZFNs), transcription activator-like effector nucleases (TALENs), and especially the clustered regularly interspaced palindromic repeats CRISPR/Cas9 systems, have improved enormously the engineering of microbes for an efficient production of target products [247,248,249,250,251,252].

The discovery of marine NPs experienced increasing success in the biotechnology field thanks to the rapid advances in systems biology and bioinformatics (see paragraph 3.1), and thanks to tools like metabolic engineering and gene editing [253] that enabled the construction of microbial cell factories. Microbial cell factories are engineered microorganisms used as production facilities to optimize biosynthetic pathways and the production of chemicals of interest from different sources [254].

In this review, we focused our attention on examples of metabolic engineering of marine native producers. For approaches of heterologous expression and metabolic engineering of heterologous hosts, we recommend to read the following reviews [255,256].

Metabolic engineering has been successfully applied to the marine environment, particularly to microalgae, and there are many examples of precious secondary metabolites, whose production was significantly improved in the native organism by metabolic engineering (Table 6).

Astaxanthin is a red, cyclic C40 carotenoid. It is known to be one of the best natural antioxidants, and it can be used for skin protection, hair growth promotion, as anti-aging, helping alleviate sports fatigue, but also for preventing cancer, cardiovascular diseases, and diabetes [257,258]. Many studies have carried out attempts of astaxanthin production by the metabolic engineering of naturally astaxanthin-producing microorganisms [259,260]. Astaxanthin is accumulated in many microorganisms such as the marine bacterium *Agrobacterium aurantiacum* [261], *Chlorella zofingiensis*, and *Chlorococcum*, and the yeast *Phaffia rhodozyma* [262,263]. However, over the years, some attempts have been carried out to increase the amount of astaxanthin naturally produced by these microorganisms. For example, the microalga *Aurantiochytrium* sp. SK4 was metabolically engineered to produce astaxanthin, by overexpressing the hemoglobin gene from the bacteria *Vitreoscilla stercoraria*, which resulted in a significant increase in the astaxanthin production by 9-fold to a final titer of 131 μg/g DCW [264]. Modification for improved astaxanthin production were also performed on bacteria, like *Paracoccus* sp. and *Sphingomonas* sp. Ide and collaborators induced random mutagenesis and the overexpression of the genes *crtW*, *crtZ*, *crtY* (encoding lycopene cyclase), *crtI*, and *crtB* (encoding phytoene synthase) to improve astaxanthin production in *Paracoccus* sp. and the fermentation of this engineered strain produced astaxanthin to a titer of 480 mg/L (8 mg/g DCW) [259].

Zeaxanthin is another carotenoid of great interest. It is known for its antioxidant properties and ability to repair photo-oxidative damage. Furthermore, zeaxanthin plays a crucial role in high visual acuity and central vision, protecting the human eyes from blue light [265]. Metabolic engineering in halotolerant marine microalga *Dunaliella salina* was performed to increase zeaxanthin production. To do so, the β-carotene hydroxylase gene from the green-alga *Chlamydomonas reinhardtii* was introduced in *D. salina* and 0.2 mg/g DCW zeaxanthin was obtained. Besides violaxanthin, another bioactive compound, improved its yield by reaching 3.13 mg/g DCW [266].

DHA and EPA are important omega-3 Poly-Unsaturated Fat Acids (PUFAs) biosynthesized by marine organisms. They are naturally present in fish and microalgae but, because of climate change, ocean pollution, and overfishing, these compounds are decreasing but the demand keeps increasing. Therefore, there is an urgent need of EPA and DHA from alternative sources [267]. DHA was suggested to be used for preventing neurodegeneration [268], and is massively present in the cerebral cortex for cognitive functions [269]. EPA and DHA have anti-inflammatory effects partially due to their inhibition of pro-inflammatory eicosanoid synthesis from ω-6 arachidonic acid [270]. In fact, they were also studied as a possible solution to treat SARS-CoV-2 (COVID-19) infection [271]. *P. tricornutum* is a unicellular marine diatom that was metabolically engineered to produce DHA. In the wild-type strain, the DHA level is very low. In addition, the co-expression of △5-elongase OtElo5 and △6-desaturase OtD6 from the unicellular species of marine green alga *Ostreococcus tauri* in *P. tricornutum* UTEX646 increased the DHA content to 11% of total fatty acids [272].

*Nannochloropsis oceanica* is a marine microalga and its total lipid content accumulates up to 60% DCW, and efforts of metabolic engineering aimed to increase EPA assembly into triacylglycerol (TAG) [273]. The malonyl-CoA:ACP transacylase was overexpressed, leading to the 8% DCW more of total lipid content and EPA than the wild-type [274]. EPA accumulation was even more enhanced by 5% due to the overexpression of Δ5 desaturase, and even up to 25% in total lipids for the co-overexpression of Δ5 desaturase and Δ12 desaturase [275]. Moreover, diacylglycerol acyltransferase DGAT2K overexpression led to 3% increase in EPA compared to a knockout mutant in nitrogen depletion conditions and 2% more than the wild-type [276]. Another example is provided by Liang et al., who attempted to increase DHA production in the thraustochytrid *Aurantiochytrium* sp. by disrupting PUFA beta-oxidation through the knockout of the 2,4-dienyl-CoA reductase gene and the constructed strain was called KO strain. The KO strain produced 57.34% more DHA than the wild-type. Furthermore, DHA production increased over time, from 63 to 90 h, from 170.03 to 203.27 mg/g DCW, whereas in the wild-type strain, DHA production decreased over time from 150.58 to 140.10 mg/g DCW [277].

The use of tools like ZF, TALENs, and CRISPR/Cas9 applied to metabolic engineering is a very promising step for the exploitation of metabolites from marine environments [278]. However, despite great progress in the fields, the hurdles for commercialization remain firm because of the complexity of marine NP structures and of their biosynthetic pathways. Above all, the titer, productivity, and yield of NPs still need to be further enhanced [246]. There are numerous bottlenecks in the biosynthetic pathways of these molecules that need further elucidation. Besides the availability of gene editing tools, detailed information about metabolism and intracellular fluxes is required to evaluate the modifications correctly. Moreover, there is the need to develop more suitable marine models to produce these bioactive metabolites. The continuous improvement of gene editing tools, correlated with a deeper understanding of marine metabolites biosynthesis, will be key to ultimately unlocking the potential of marine molecules for biotechnology.

**Table 6:** List of molecules obtained with metabolic engineering of native organism for higher yield.

Microorganism	Class	Metabolite Type	Yield Increment	References
		**BACTERIA**		
*Paracoccus* sp. N-81106	Terpenoids	Astaxanthin	1700%	[259]
*Sphingomonas* sp. ATCC 55669	Terpenoids	Astaxanthin	540%	[279]
		**CYANOBACTERIA**		
*Synechocystis* sp. PCC 6803	Terpenoids	Squalene	300%	[280]
		**MICROALGAE**		
*Aurantiochytrium* sp.	Terpenoids	Astaxanthin	900%	[264]
*Dunaliella salina*	Terpenoids	Zeaxanthin	200%	[266]
*Phaeodactylum tricornutum*	PKS	DHA	800%	[272]
*Aurantiochytrium* sp.	PKS	DHA	57.34%	[277]
*Nannochloropsis oceanica*	PKS	EPA	8%; 5%; 3%	[275]

PKS: polyketide synthase; DHA: docosahexaenoic acid; EPA: eicosapentaenoic acid.

## 6. Linking the Biotechnological Potential of Marine Microorganisms with the Blue Economy Sector

Biotechnology can benefit from many microbial resources and, concerning the marine biome, several microbes are now in use in the Blue Economy skyline due to their biotechnological potential [281], but many others have still to be exploited. The microbial applications range from carbon dioxide sequestration and mitigation of global climate change to the production of biomass and valuable compounds with no need for agricultural land.

In many cases, marine bacteria show remarkable biotechnological potential, producing commercially valuable products such as surfactants, glyco- and lipopeptides, exopolysaccharides, and pigments that are also endowed with several bioactivities, including antimicrobial, antiproliferative, antioxidant, and anti-inflammatory properties. For example, marine bacteria are producers of various pigments, e.g., melanin, violacein, phenazine, carotene, prodigiosin, and quinone, that often exhibit antimicrobial, antifungal, antiproliferative, photoprotective, antiparasitic, and immunosuppressive activities.

Among biosurfactants, surface-active compounds with environmental and industrial applications, many marine bacteria are reported to produce valuable mixtures of rhamnolipids, e.g., *Pseudomonas gessardii* [282,283], *Shewanella aquimarina* [2], *Rhodococcus* sp. [67,284]. In addition, bacterial toxins can be exploited as antifouling agents for the production of antifouling coatings, as in the case of the metabolite taxifolin isolated from *Streptomyces sampsonii* PM33 [285]. However, their marine origin is often lost when transformed into products for the detergent market.

Equally, marine-derived enzymes show great potential for different biotechnological applications, e.g., the production of green bioenergy from vegetal biomass waste [286,287] thanks to their biocompatible, eco-friendly, and high effectiveness characteristics. For a wide range of industrial applications, the utilization of enzymes derived from microorganisms is preferred over those derived from non-microorganisms due to their comparatively lower production costs, easy access to raw materials, rapid growth, and well-defined cell metabolism. A recent review describes the different types and sources of marine enzymes [288].

In addition, marine bacteria are known to be able to interact with different types of pollutants, defining their potential for bioremediation purposes [289,290,291,292,293,294]. Also, they can be employed as biofertilizers and bio-stimulants with exceptional pesticide activities, plant growth regulators, and seed-coated fungicides [295]. Bacteria producing biomaterials and biogenic nanoparticles are also of great interest to the manufacturing sector [296]. As a raw resource, marine biopolymers present new prospects for the development of bio-based materials in response to the growing need for biodegradable plastics to reduce plastic pollution in marine environments [297,298]. Lastly, marine bacteria can also find applications in other fields, such as pharmaceutical for drug delivery [299,300], environmental biotechnology for pollutant removal [301], and bio-metallurgy for the recovery of valuable elements and production of secondary raw materials [302]. Velmurugan and coauthors provide a good perspective on their applications in different fields [303].

Similarly, the kingdom of fungi also accounts for many marine strains with exceptional biotechnological potential. In this regard, in the previous paragraphs, our review highlights the potential of marine fungi as producers of bioactive molecules with different application fields thanks to their antioxidant, anti-inflammatory, antibacterial, antiviral, and antiproliferative properties. A detailed description of the antioxidant properties of marine fungi is given by Vitale et al. [304], highlighting their extraction protocols and industrial applications. Bioactive compounds derived from marine fungi are also widely used for the development of new cosmeceuticals with anti-aging, wound healing, and skin whitening functions, or used against skin infections [305]. Moreover, a recent work showed the lipogenesis capacity of *Paradendryphiella* sp. in acidic/alkaline and psychrophilic/mesophilic conditions, and its capacity to inhibit the proliferation of different cancer cell lines [306].

Marine fungi are also employed in the food and feed industry. In the first case, an example is given by *Paradendryphiella salina* used to increase the nutritional value of *Macrocystis pyrifera* and seaweed waste within a fermentation process for the production of mycoprotein [307]. In the aquaculture sector, feeds also show increased nutritional values when fermented with marine fungi, as in the case of catfish fed with a marine fungal-fermented diet showing a consistent improvement in their growth [308].

The vast potential of these microorganisms also has promising applications in the bioremediation field, as they are known to accumulate heavy metals and degrade various pollutants, among which is a wide range of complex hydrocarbons [309,310]. For example, *Penicillium polonicum* AMF16, *Penicillium chrysogenum* AMF47, and *Penicillium cyclopium* AMF40 and AMF74 could be grown using crude oil as the sole carbon source, and produce high amounts of biosurfactants, though not yet characterized [311]. They are widely used for wastewater treatments. Some fungal species can also synthesize biopolymer-degrading enzymes (i.e., hydrolases, lyases, oxidoreductases) [297], and thanks to their efficient enzymatic profile, are primary decomposers of natural polymers including cellulose and lignin [312]. In addition, fungal strains belonging to the families *Dipodascaceae*, *Microascaceae*, *Gymnoascaceae*, and *Trichocomaceae* isolated from highly contaminated coastal sediments are able to bioaccumulate precious metals from electronic waste (e.g., platinum, palladium, silver, and gold) [313]. Some examples of industrial applications of yeast and fungi marine-derived enzymes were recently reported [288].

Marine microalgae are other key players of the biotechnology industry, which has become a lucrative global business, valued in the multi-billion-dollar range. The industry produces a diverse range of commercial products [314,315]. However, despite the enormous microalgal diversity [316], few strains are currently commercialized for the production of high-value products.

Marine microalgae are characterized by high protein content, PUFAs, enzymes, polymers, lipids, peptides, vitamins, toxins, sterols, and a set of pigments that are attractive to many economic sectors, such as health, nutrition, and cosmetics. Moreover, their biomasses gain value through the development of beneficial metabolites [317].

Many compounds are known to have antioxidant, antiproliferative, antimicrobial, and anti-inflammatory properties. For example, the marine diatom strain of *Cylindrotheca closterium* showed such properties [318]. Equally, a research study on *Tisochrysis lutea* investigated its therapeutic activity against dry eye syndrome (DES), demonstrating that it can reduce inflammation on ARPE-19 human retinal pigmented epithelial cells when used as functional food [319]. For more examples related to these industrial sectors, please refer to the reviews of Zhuang et al. [320].

Microalgae-based food and feeds are often used because of their proven nutritional value and digestibility. The human health sector recognizes diatoms as the most valuable source of primary and secondary metabolites. Of great interest because of their direct extraction procedures are chrysolaminarin, EPA and DHA, and fucoxanthin. *P. tricornutum* is now among the most researched and characterized diatom species. It is also the source of an EPA-rich oil that is currently being evaluated by the European Food Safety Authority (EFSA) in Europe and is already being used as a supplement in the USA [321]. For the development of plant-based feeds, DHA yield was maximized in *Aurantiochytrium* sp. by applying salt stress and reusing a sugar refinery washing water (SRWW) waste [322]. The same genus was also mutagenized, achieving a mutant strain with increased biomass, lipid, and DHA, compared to the wild-type strains [323]. In Section 5, more examples of metabolic engineering that led to increased DHA yields in microalgae are reported.

Among the many valuable compounds, lipids are of great interest to the energy sector; therefore, biofuels from microalgae appear as an alternative to conventional fossil fuels, clearly more sustainable and environmentally friendly but still with a large margin for improvement [324]. As a matter of fact, cultivation, harvest, oil extraction, and conversion of microalgal lipids are the main steps in manufacturing biofuels from microalgae biomass since harvesting and oil extraction account for 60% of the cost of biofuel production [325]. In-depth studies on the lipogenesis process were also reported [326]. Moreover, the review by Sharma et al. 2018 gives an interesting overview of the genetic manipulation of the main genes mutated for oil enhancement [324].

Microalgae also have interesting potential in bioremediation applications. For example, *Chlorella marina*, *Tetraselmis suecica*, and *Picochlorum maculatum* are able to remove inorganic nitrogen and phosphorus from wastewater, even when immobilized in alginate [327]. *P. tricornutum* was used to create a microbial cell factory to produce and secrete an engineered form of PETase into the surrounding culture medium useful to degrade PET under mesophilic growth conditions [328]. Moreover, four microalgal species (*Phaeocystis globosa*, *Nannochloropsis oculata*, *D. salina*, and *Platymonas subcordiformis*) were able to biodegrade an endocrine disruption compound (EDC), with efficiencies ranging from 43 to 90.9% [329], while *Rhinomonas reticulata* showed potential in the bioremediation of propylbenzenes (an alkylbenzene widely used in fuel that causes water oil pollution) [330].

### Blue Biotechnology Market and Shareholders

Blue biotechnology is defined by the Organization for Economic Co-operation and Development (OECD) (2016) as the “application of science and technology to living aquatic organisms for the production of knowledge, goods and services”. Hurst and colleagues [331] further define it as a multi-disciplinary, knowledge, and capital-intensive technology. It is difficult to categorize the newly emerging sector of blue biotechnology clearly by the usual classification of business activities. A possible reason is the complex network of domains and activities that characterize the sector, most of which are strictly linked to research and product development. Moreover, the blue biotechnology value chain comprises steps at different maturity levels and diverse technological stages, performed by different actors. This means that “*the blue biotechnology sector is research-intensive and driven by innovative activities carried out by innovative SMEs*, *smaller service companies and research infrastructure*, *but with relatively low involvement of large companies*” [332]. Its market segmentation can change according to the standpoint, i.e., sources, technologies, applications, end-user industries, and even based on geographic regions. From this perspective, it is easy to explain the slim number of companies operating in the blue sector, especially by adding a filter for those working only with marine microbes. Moreover, biotech companies frequently lose the “blue” component of their source when achieving their final product.

The list of the companies, small and medium-sized enterprises (SMEs), consortia, and start-ups/spin-offs reported in Table 7 are some of the leading players operating in the Global Marine Biotechnology Market, also found with the help of the European Union Bio-based Industries consortium (https://biconsortium.eu/membership/full-members accession date 8 October 2024) and the review of Rotter et al. 2021 [281].

**Table 7:** A list of the companies, SMEs, consortia, and start-ups/spin-offs operating in the Global Marine Biotechnology Market. URLs have been accessed on 6 November 2024.

Company	Website	Country	Product or Company Description
Greensea	http://greensea.fr/en/	France	Culture of marine and freshwater microalgae for applications in the food, cosmetic, and health markets.
CODIF	https://www.codif-tn.com/en/	France	Cosmetic product Dermochlorella DG^®^.
GreenTech	https://www.greentech.fr/en/	France	Cosmetics from marine microalgae
AlgoSolis	https://algosolis.com/en/	France	Microalgae R&D facilities.
Mycrophit	http://microphyt.eu/	France	Production of unique microalgae-based bioactive compounds using breakthrough technologies and processes.
Consorzio Italbiotec	https://www.italbiotec.it/index.php/en/	Italy	Public–private consortium focused on industrial biotechnology and bioeconomy. Italbiotec brings together eight universities and 48 institutions and companies.
Microperi Blue Growth	https://www.micoperibg.com/	Italy	Microalgae cultivation for nutraceutical, pharmaceutical, medical, agricultural applications.
TERE GROUP	https://www.teregroup.it/omega-3/	Italy	Cultivation of *Crypthecodinium cohnii* for omega-3 production.
PriGen Srl	https://genhyalskincare.com/en/	Italy	Skincare branded product Gen-Hyal^®^
Fitoplancton Marino	http://www.fitoplanctonmarino.com/en/index.html	Spain	Production and commercialization of products with high value-added derived from marine microalgae.
Plancton Marino	https://www.planctonmarino.com/en/	Spain	Food ingredients from marine microalgae.
MONZON BIOTECH		Spain	Produces two different microalgae species, *Nannochloropsis* for aquaculture larval feed and *Dunaliella salina* for cosmetic applications.
NEOALGAE	https://neoalgae.es/?lang=en	Spain	Food, cosmetics, agro-industrial products from marine microalgae.
Lipotec (Lubrizol Corporation)	https://www.lubrizol.com/Personal-Care	Spain	Ingredients and polymers for cosmetics from marine bacteria and yeast.
PharmaMar	https://pharmamar.com/?lang=en	Spain	Molecules with anticancer activity.
Allmicroalgae	https://www.allmicroalgae.com/en/microalgae/	Portugal	Food, feed, cosmetics, agro-industrial products from marine microalgae.
a4f algae for future	https://a4f.pt/en	Portugal	Specialist company in most aspects of microalgae production.
BIOALVO S.A.	bioalvo.com	Portugal	Drug discovery and products to address healthcare needs.
Bluevert	https://bluevert.com/en/	Portugal	Cosmetics marine microalgae.
Biotrend	https://www.biotrend.pt/	Portugal	Services in the area of industrial and marine biotechnology, specialized in the development, optimization, and scale-up of bioprocesses.
Lonza Group AG	https://www.lonza.com/	Switzerland	Pharmaceutical and ingredients from marine microorganisms.
Alganex	https://alganex.com/de_DE/	Germany	Microalgae-based products distributor.
Ocean Basis	https://www.oceanbasis.de/en	Germany	Production of natural substances from marine microorganisms used for food, nutraceuticals, and cosmetics.
ALIGA	https://www.aliga.dk/	Denmark	Nutraceutics from microalgae (among which is *Schizochytrium* sp. for DHA).
Royal DSM N.V.	https://www.dsm.com/corporate/home.html	Netherlands	Aquaculture’s feed DHA and EPA rich.
ArcticZymes Technologies	https://www.arcticzymes.com/	Norway	Research on arctic marine microorganisms for the discovery of cold-adapted enzymes for industrial applications improving high-salt and low-temperature efficiency.
Prolume Ltd.	https://www.prolume.com/	USA	Genes from deep water marine bioluminescent organisms. This has broad applications for biomedical research, drug discovery, and entertainment.
Reed Mariculture	https://reedmariculture.com/	USA	Feed production for aquaculture.
Microalgae (Proviron Company)	https://proviron.com/microalgae/	USA–China–EU	Feed production for aquaculture.
Xiamen Huison Biotech Co., Ltd.	http://www.chinahuison.com/EN/index.aspx	China	Production of lyophilized biomass of *Schizochytrium* sp. DHA rich.
Frutarom Industries Ltd.		Israel	Cosmetic Alguard^TM^ from red microalgae *Porphyridium cruentum.*

It has been difficult for some companies to identify the original marine source of the product, sometimes due to trade secrets but primarily when the product is derived not from the whole microorganism, but only from its proteins/enzymes and metabolites/active compounds. For example, other companies that probably market products of marine origin (but it is not clearly specified in the final product) are Algaenergy (https://www.algaenergy.com/ accessed on 6 November 2024) located in Spain, producer of bio-stimulants for agronomic applications; Novozymes (https://www.novozymes.com/en accessed on 6 November 2024) in Denmark, producer of enzymes for industrial applications; and TerraVia Holdings, Inc. (formerly Solazyme) (https://www.algenist.com/) a USA company and producer of a plethora of different products.

Currently, many of the NPs on the market are linked to the pharmaceutical industry. There are 17 marine-derived bioactive compounds available up to date and approved by the European Medicines Agency (EMA) and Food and Drug Administration (FDA) for clinical use [333]. Every two years, the authors publish “Marine Compounds and Cancer: Updates” in the homonym topical collection, giving a systemic review of recent progress and updates on marine-derived drug discovery. The authors, in the updates of 2020, stated that besides the approved drugs, 23 other molecules were in different stages of clinical development [334]. Most of the marine NPs in use (c.a. 71%) are drugs for cancer therapy, many of which are antibody–drug conjugates [335]. Still, some of them cure chronic pain, viral infections, and hypertriglyceridemia, while others undergoing phase I-II-III clinical trials have clinical use for Alzheimer’s, obesity, inflammation, schizophrenia, and amyloidosis [335,336].

The Marine Biotechnology Market is relatively young, but it has a promising future thanks to the increasing interest of the EU policy and consumer demand, and the research-intensive and innovative activities are providing lots of interesting in possible applications for the market. Despite the promising growth prospects, the marine biotechnology sector has to face different challenges: (i) the lack of biotechnology-related investment in research and development is limiting the market’s growth; (ii) expensive and long-term discovery processes; and (iii) strict government regulations, although the EU support policy instrument is favoring the discovery of biotechnological innovation potential. In conclusion, all the examples mentioned in this review with all the possible applications of marine microorganisms could represent a key point for the growth of the blue bioeconomy.

## 7. Conclusions

Marine microorganisms represent an important frontier in the search for bioactive NPs, offering wide potential for innovation. Over the past decade, efforts to overcome challenges in accessing and analyzing marine-derived microbial compounds have yielded important advances. The combination of cutting-edge tools and interdisciplinary strategies has not only expanded the range of metabolites discovered but has also deepened our understanding of their biosynthetic origins and ecological roles. For this reason, marine microorganisms appear to have a key role in the global bioeconomy, particularly in the rapidly expanding Blue Economy, with marine biotechnology representing a significant and growing segment. The major innovations in the use of marine microorganisms are expected to drive this growth, contributing to the development of high-value compounds for diverse applications. This review aims at contributing to this goal, providing a snapshot of the last 10 years on the status of NP discovery from marine microorganisms, and the approaches that brought to their discovery. However, it is worth noting that despite the progress in genomics and metabolomics, most of the bioactive molecules identified so far are mainly derived from cultivated microorganisms. For this reason, innovation in isolation and cultivation strategies remains at the moment crucial for the marine biodiscovery pipeline.

Integrating marine-derived products into commercial pipelines requires continued investment in scalable production systems, regulatory frameworks, and partnerships between academia and industry. The contribution of literature reviews in disseminating the scientific results helps keep the dialog and collaborations active among all the actors. It is worth noting the efforts of the EU to favor the development of new products and applications from marine bioresources, which can be used in energy, health, nutrition, cosmetics, environment, agriculture, and manufacturing sectors.

## Data Availability

Not applicable.
